# Supplementary material for: Protective near-germline antibodies to malaria elicited by RTS,S/AS01E vaccination in African infants
Source: bioRxiv. 2026 Oct 4:2026.10.01.755937. Preprint. [Version 1] doi: 10.64898/2026.10.01.755937 (PMC13648702; doi:10.64898/2026.10.01.755937)
Supplement: Supplementary file 1 [file media-1.pdf]

Supplementary Materials for

Protective near-germline antibodies to malaria elicited by RTS,S/AS01$_E$ vaccination in African infants

Emma Bloch, Fiona Devys, Fabien Cannac *et al*.

Corresponding authors: Andrew B. Ward, andrew@scripps.edu; Rogerio Amino, rogerio.amino@pasteur.fr; Gemma Moncunill, gemma.moncunill@isglobal.org, Carlota Dobaño, carlota.dobano@isglobal.org; Luis M. Molinos-Albert luis.molinos@isglobal.org

The PDF file includes:
Figs. S1 to S18
Tables S1 to S8

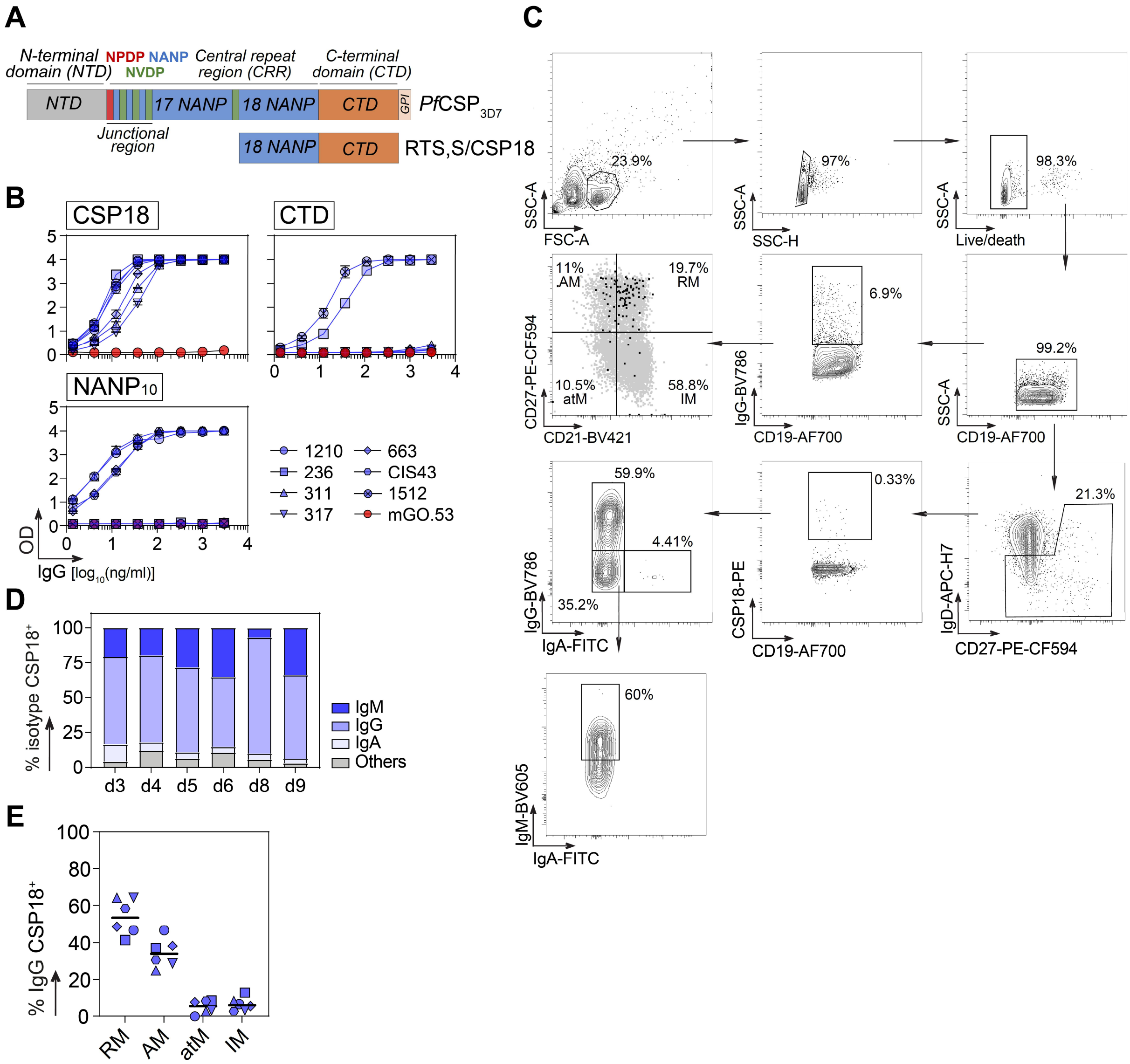

Figure S1. **CSP18 antigenicity and memory B cells from infants immunized with RTS,S/AS01$_E$.** (**A**) Sequence diagram of the *Pf*CSP protein (top) and the RTS,S/CSP18 protein construct (bottom). (**B**) ELISA titration graphs showing the reactivity of benchmarked *Pf*CSP antibodies (blue symbols), and a negative control (mGO.53, red circles) to CSP18, NANP$_{10}$ and CTD proteins. (**C**) Representative flow cytograms showing the gating strategy used to identify circulating blood CSP18-reactive IgG$^+$ B cells prior to single cell sorting, and memory B-cell (MBC) analysis. (**D**) Bar graph showing the isotype distribution of CSP18-reactive MBC for each donor (d). (**E**) Graph showing the percentage of CSP18-reactive IgG$^+$ MBC according with their phenotype as defined in (C). RM, resting memory, AM, activated memory, atM, atypical memory, IM, intermediate memory.

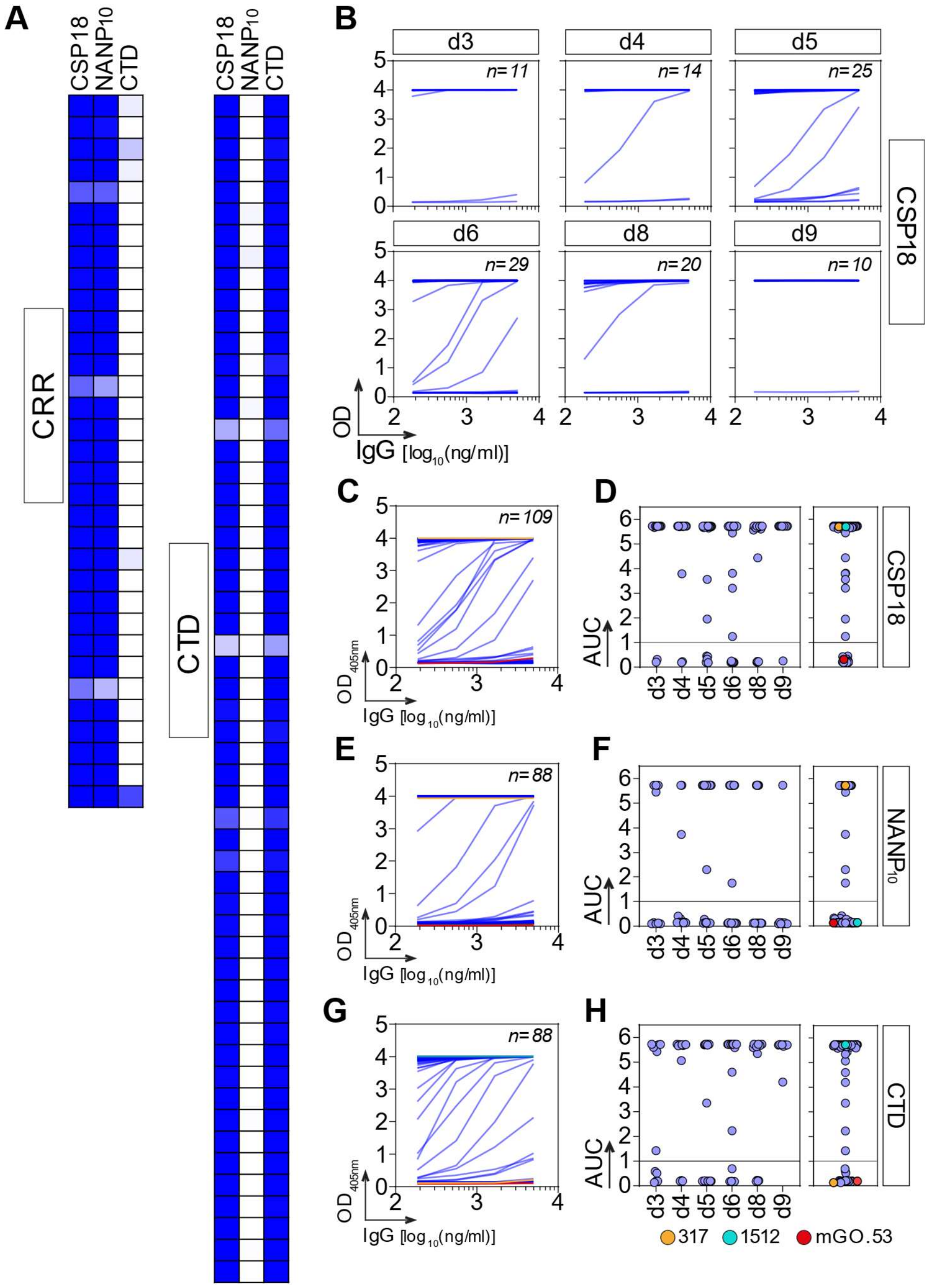

Figure S2. **Reactivity of CSP18 IgG B-cell antibodies**. (**A**) Heatmap showing the reactivity of CSP18-reactive antibodies (*n*=88) against CSP18, $NANP_{10}$ and CTD proteins, with color-coded cells according to the values shown in Table S2. The heatmap is segregated according to antibody specificity to the central repeat region (CRR, left) or the C-terminal domain (CTD, right). (**B**) Representative ELISA titration graphs showing the reactivity of antibodies cloned from $CSP18^+$ B cells. The number of tested antibodies is indicated in italics. (**C**) Same as in (B) but for all cloned antibodies together. (**D**) Dot plots showing the area under the curve calculated from graphs in (C), segregated by donors (left) or together (right). (**E-F**) Same as in (C-D), respectively, but for reactivity against the $NANP_{10}$ protein. (**G-H**) Same as in (E-F), respectively, but for reactivity against the CTD protein. The number of antibodies are indicated in italics. Positive controls for $NANP_{10}$ (317, orange) and CTD (1512, cyan), and negative isotype control (mGO.53, red) are indicated.

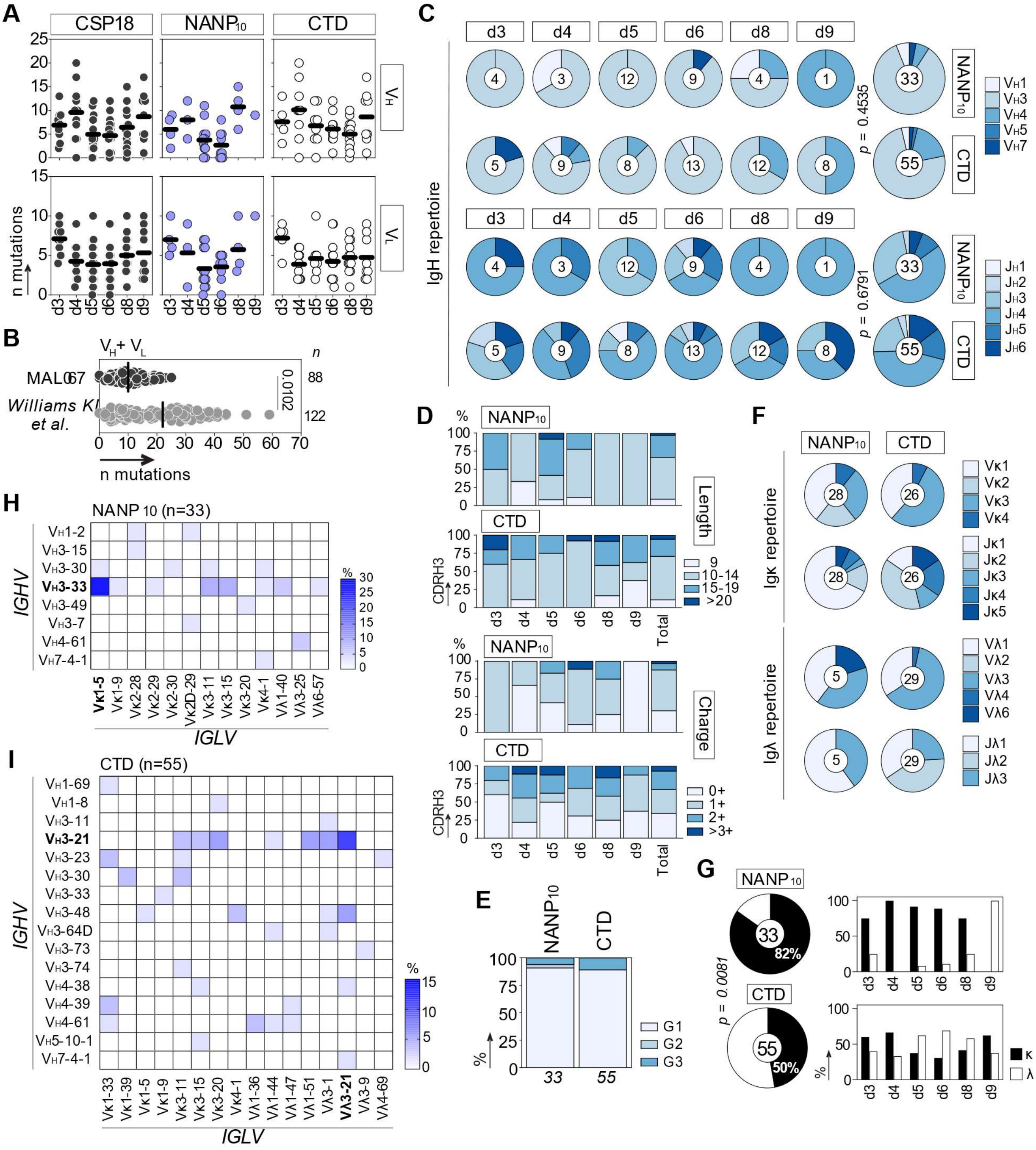

Figure S3. **Immunoglobulin gene repertoire of CSP18 IgG B-cells**. (**A**) Dot plots showing the number of nucleotide mutations in V_H (top) and V_L (bottom) genes of all the CSP18 B-cell antibodies (n=88, black), or grouped by $NANP_{10}$ (n=33, blue) and C-terminal domain (CTD, n=54, white) reactivity. Averaged mutation per donor is indicated by the solid black lines. (**B**) Dot plot comparing the number of somatic mutations in V_H+V_L genes of all CSP18 antibodies from our collection, and *Pf*CSP antibodies cloned from RTS,S-vaccinated adults (data from *Williams KL et al, (2024), Nat. Med. 30:117-129, Doi: 10.1038/s41591-023-02659-z*). Groups were compared with the two- tailed Mann- Whitney test. *p* value is indicated. (**C**) Pie charts comparing the distribution of V_H and J_H gene usage of $NANP_{10}$ and CTD B-cell antibodies. The number of antibody sequences analyzed is indicated in the center of each pie chart. (**D**) Bar graph showing the distribution of amino acid length (top) and

total positive charges (bottom) of the CDRH3 of $NANP_{10}$ and CTD B-cell antibodies. (**E**) Bar graph showing the distribution of IgG subclass of $NANP_{10}$ and CTD B-cell antibodies. (**F**) Same as in (C) but for the Igk and Igλ repertoire. (**G**) Pie chart (left) and bar graphs (right) comparing the distribution of kappa vs lambda Ig chain usage between $NANP_{10}$ and CTD B-cell antibodies. (**H**) Heatmap showing the frequency of $NANP_{10}$ B-cell antibodies encoded by the indicated IGHV and IGHL pairs. (**I**) Same as in (H) but for CTD B-cell antibodies. Groups were compared using 2x5 Fisher's Exact test in (C) and (G).

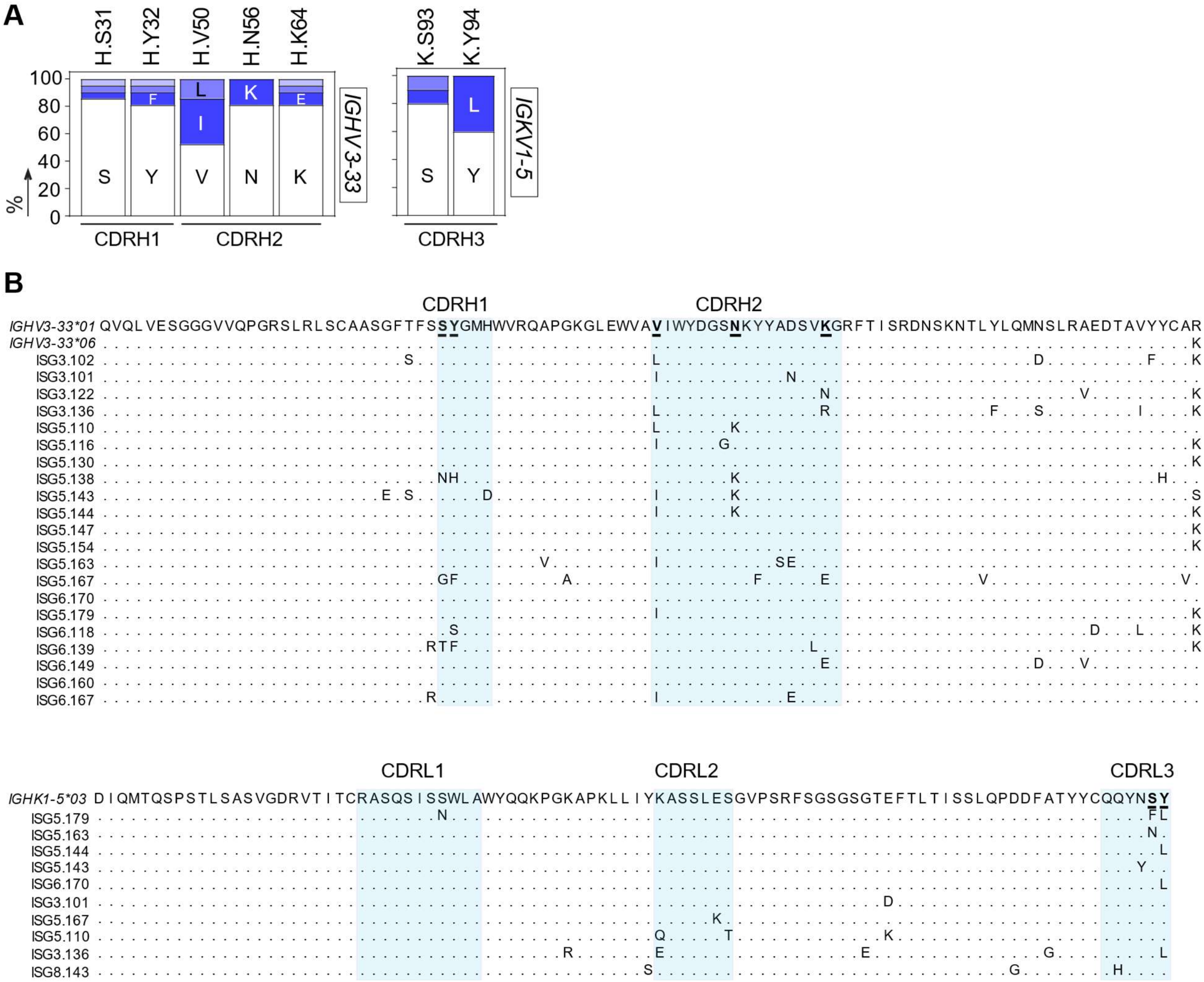

Figure S4. **Amino acid replacements and alignment of *IGHV3-33/IGK1-5* NANP$_{10}$ antibodies.** (**A**) Bar graphs showing the proportion of amino acid replacements at the indicated positions of *IGHV3-33* (left) and *IGK1-5* (right) gene segments of NANP$_{10}$ antibodies. Amino acids of unmutated genes are indicated in white and replacements are indicated in shades of blue. (**B**) Amino acid sequence alignment of *IGHV3-33* (top) and *IGK1-5* (bottom) gene segment alleles of NANP$_{10}$ antibodies.

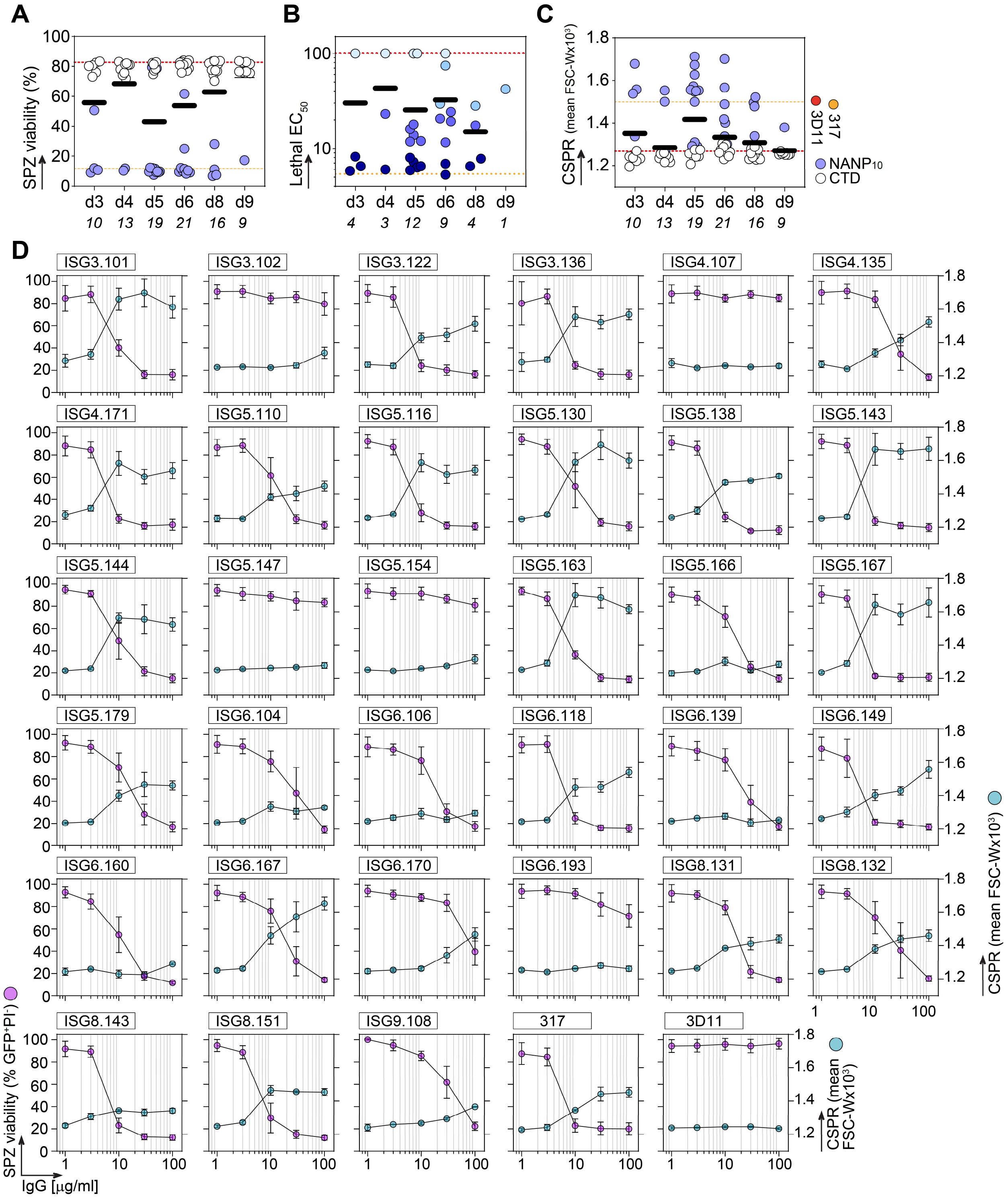

Figure S5. *In vitro* antiparasitic properties of CSP18 antibodies. (**A**) Dot plot comparing the Fab-dependent lethality induced in sporozoites (SPZ) by $NANP_{10}$ and C-terminal domain (CTD) antibodies at 100 µg/ml, after 45 min of incubation at 37ºC, segregated by donor (d), tested in duplicate in two independent experiments. (**B**)

Dot plot showing the lethal half-maximal effective concentration (EC_{50}) values induced in SPZ by $NANP_{10}$ antibodies (n=3 independent experiments, see [D]). EC_{50} values color coded in shades of blue, with darker shades indicating higher potency. (**C**) Same as in (A), but for the circumsporozoite precipitation reaction (CSPR). (**D**) Graph depicting the distinct *in vitro* lethality (purple) and CSPR (cyan) induced in SPZ by $NANP_{10}$ antibodies after 45 min of incubation at 37ºC (n = 3 independent experiments, data are presented as mean ± SEM).

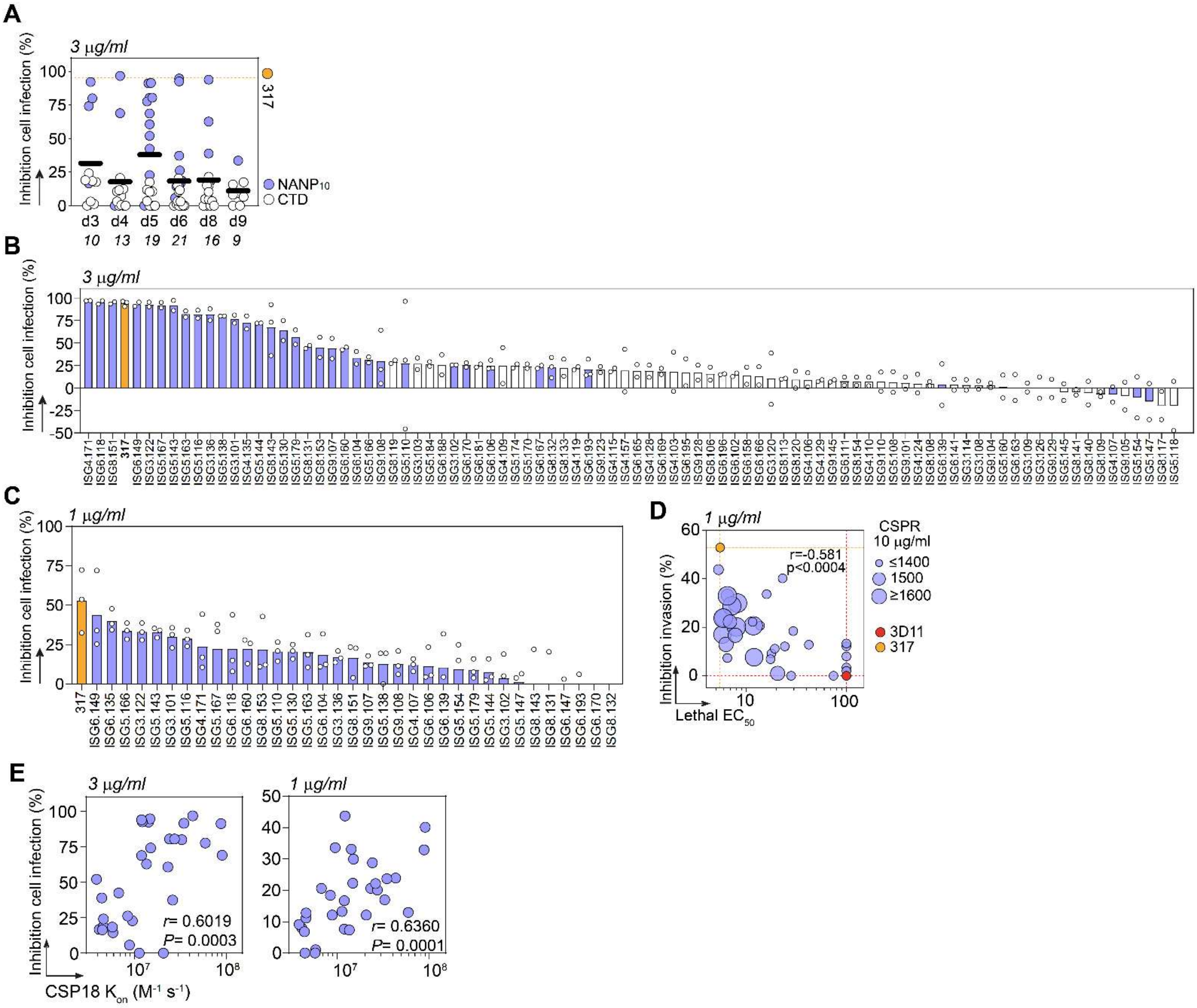

Figure S6. ***In vitro*** **inhibition of hepatocyte invasion by CSP18 antibodies**. (**A**) Dot plot comparing the percentage of sporozoite (SPZ) inhibition to invade HepG2 cells induced by $NANP_{10}$ (blue) and CTD (white) antibodies, after two hours of incubation at 37ºC, tested at 3 µg/ml in 2-3 independent experiments, segregated by donor. (**B**) Bar graphs showing the percentage of SPZ inhibition to invade HepG2 cells induced by $NANP_{10}$ (blue) and CTD (white) antibodies for each antibody tested at 3 µg/ml in 2-3 independent experiments. Each dot represents the value of an independent experiment. (**C**) Same as in (B), but for $NANP_{10}$ antibodies after 48h of incubation at 37 ºC, tested at 1 µg/ml in three independent experiments. (**D**) Bubble plot showing the lethal half-maximal effective concentration (EC_{50}) values versus the percentage of inhibition of HepG2 invasion induced by $NANP_{10}$ antibodies after 48 h of incubation at 37ºC, tested at 1 µg/ml. The size of the bubbles is proportional to the elongated CSPR phenotype induced by the antibodies at 10 µg/ml. (**E**) Correlation plots showing the association constant (K_{on}) to CSP18 versus the percentage of inhibition of HepG2 invasion of $NANP_{10}$ antibodies tested at 3 (left) and 1 (right) µg/ml. The values for the *Pf* inhibitory 317 (orange), and the *Pb* 3D11 (red) negative control antibodies are indicated in colored dashed lines or dots. The two-sided Spearman rho correlation coefficients and corresponding *p* values are shown in (D) and (E).

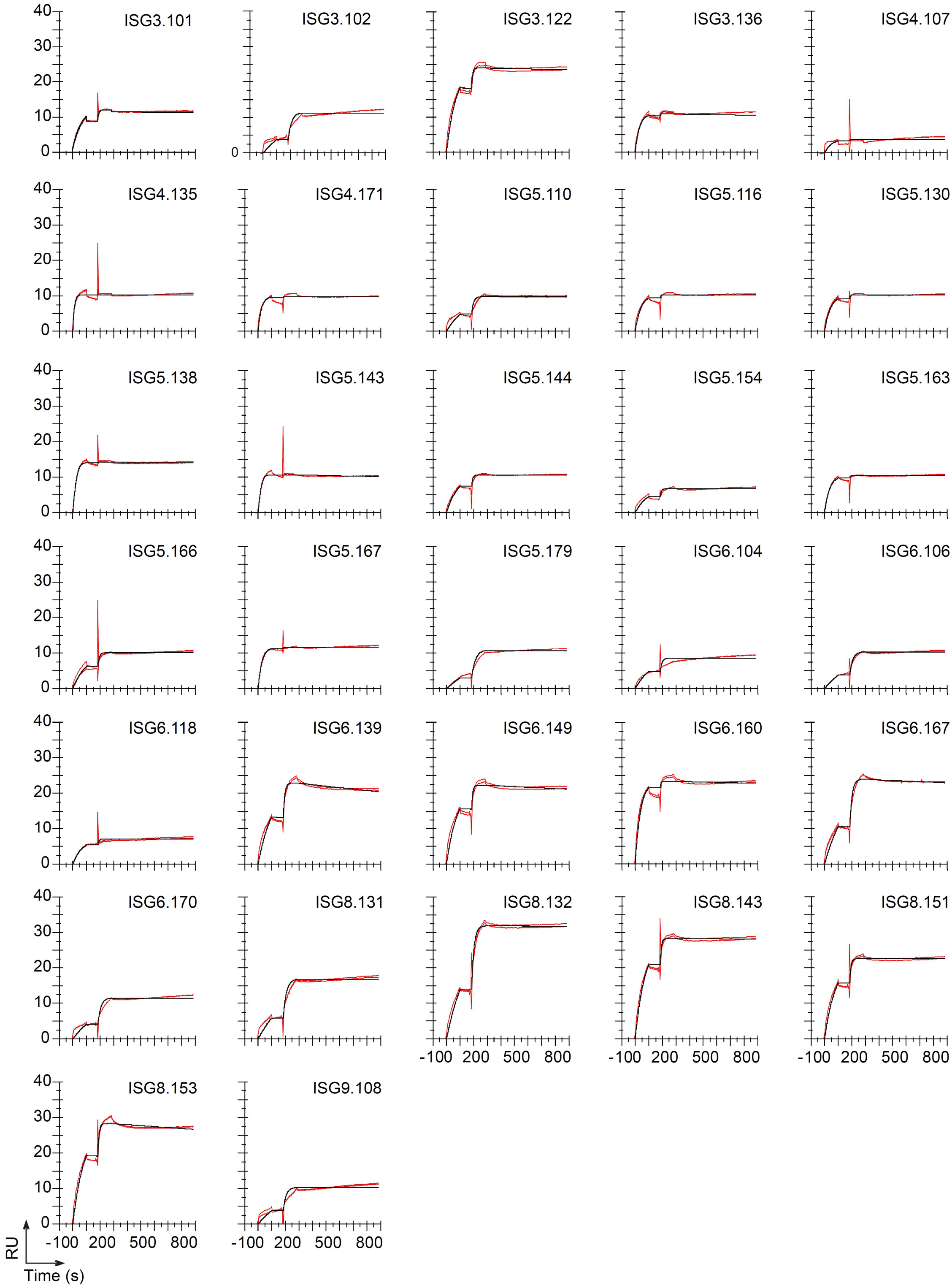

Figure S7. Surface plasmon resonance (SPR) of $NANP_{10}$ antibodies. Sensorgrams showing the single-cycle kinetics (SCK) of $NANP_{10}$ antibodies binding to CSP18.

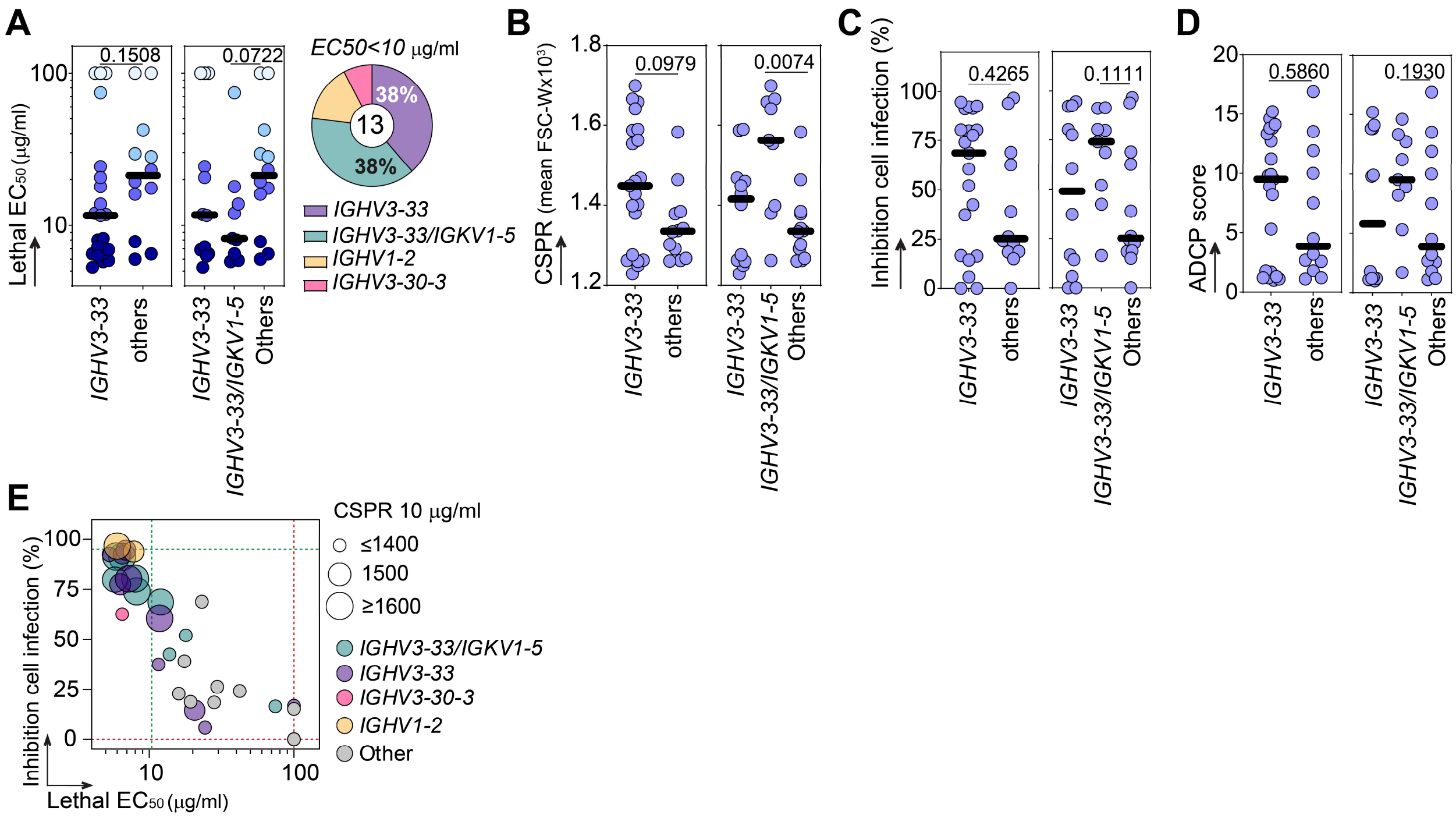

Figure S8. ***In vitro* antiparasitic properties of *IGHV3-33* NANP$_{10}$ antibodies**. (**A**) Dot plots comparing the lethal half-maximal effective concentration (EC$_{50}$) values induced in sporozoites(SPZ) by NANP$_{10}$ antibodies (n=3 independent experiments) between those encoded by *IGHV3-33* (left), matched *IGHV3-33/IGKV1-5* (right), and other gene segments. The dots are coded in shades of blue, with darker shades indicating higher lethal EC$_{50}$ potency, as indicated in Fig 2B. The pie chart shows the distribution of NANP$_{10}$ antibodies with EC$_{50}$ values below 10 µg/ml, according with the color coded *IGHV* gene family used. (**B**) Dot plots as in (A) but for the CSPR measured at 10 µg/ml. (**C**) Dot plots as in (A) but for the percentages of inhibition of cell infection, measured at 3 µg/ml. (**D**) Dot plots as in (A) but for the ADCP score. (**E**) Bubble plot showing the lethal EC$_{50}$ values versus the percentage of inhibition of cell infection induced by NANP$_{10}$ antibodies, with color coded *IGHV/IGHL* gene families. The size of the bubbles is proportional to the elongated CSPR phenotype induced by the antibodies at 10 µg/ml.

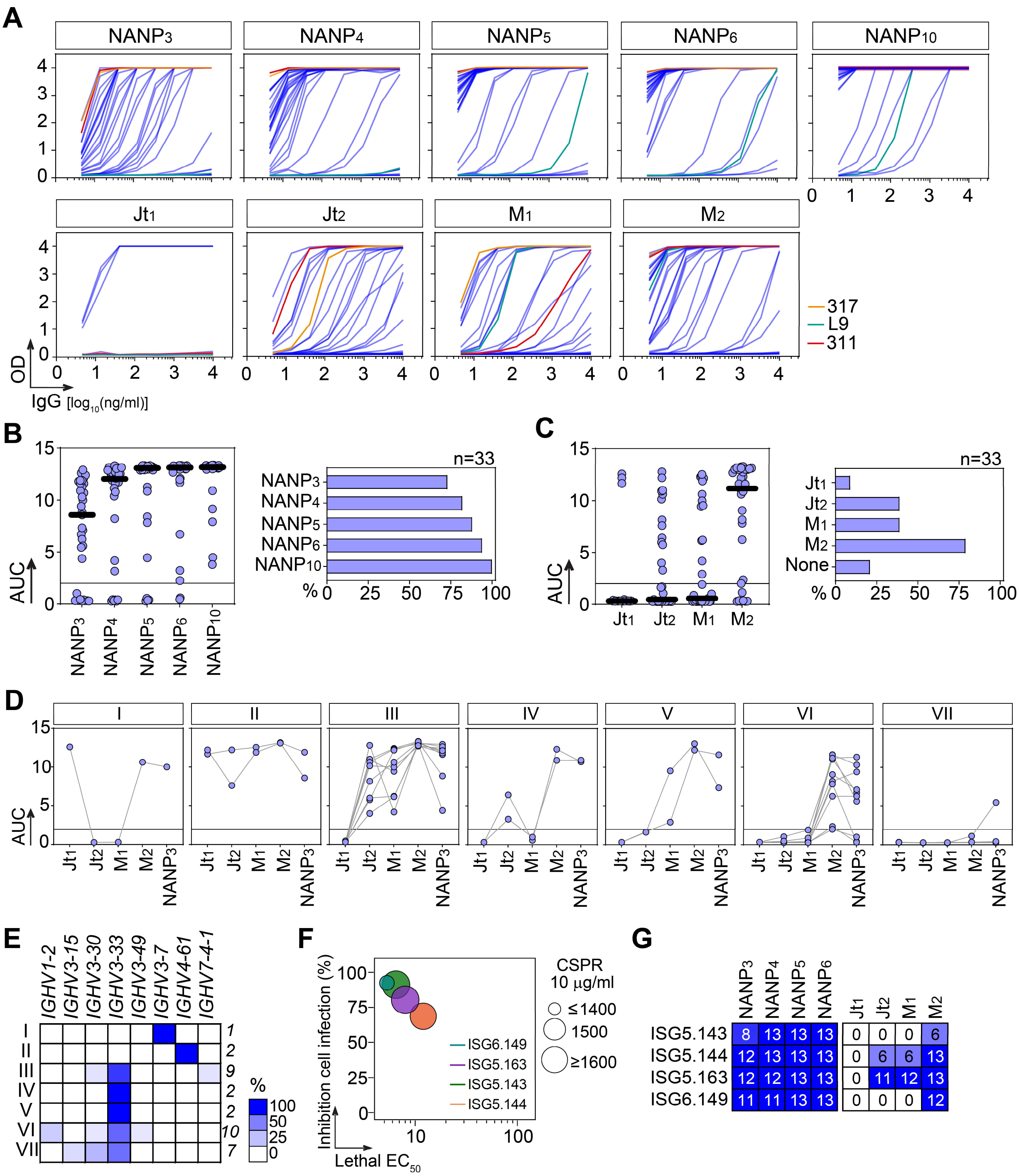

Figure S9. **Binding profiles of central repeat region (CRR) antibodies**. (**A**) Representative ELISA titration graphs of three independent experiments tested in duplicate, showing the reactivity of NANP$_{10}$ antibodies against peptides with incremental NANP repetitions (NANP$_{3-10}$, top) and peptides Jt$_1$, Jt$_2$, M$_1$ and M$_2$ (bottom). Central (311 and 317) and minor (L9) repeat control antibodies are shown. (**B**) Dot plot (left) showing the area under the curve (AUC) calculated from the ELISA graphs in (B) against NANP$_{3-10}$. The bar graph shows the proportion of antibodies recognizing each peptide. (**C**) Same as in (B) but for peptides Jt$_1$, Jt$_2$, M$_1$ and M$_2$. (**D**) Graphs showing the ELISA AUC of antibodies against the indicated peptides, grouped by different binding profiles (I-VII). (**E**)

Heatmap showing the proportion of *IGHV* gene segments used by the antibodies grouped by binding profiles I-VII from (E). (**F**) Bubble plot showing the lethal half-maximal effective concentration (EC_{50}) values *versus* the percentage of inhibition of cell infection induced by antibodies ISG5.143, ISG5.144, ISG5.163 and ISG6.149. The size of the bubbles is proportional to the elongated CSPR phenotype induced by the antibodies at 10 µg/ml. (**G**) Heatmap showing the ELISA AUC values of ISG5.143, ISG5.144, ISG5.163 and ISG6.149 against $NANP_{3-6}$ and Jt_1, Jt_2, M_1 and M_2 peptides. The color-coded cells are expressed according to the ELISA area under the curve (AUC) values with darker colors representing higher reactivity.

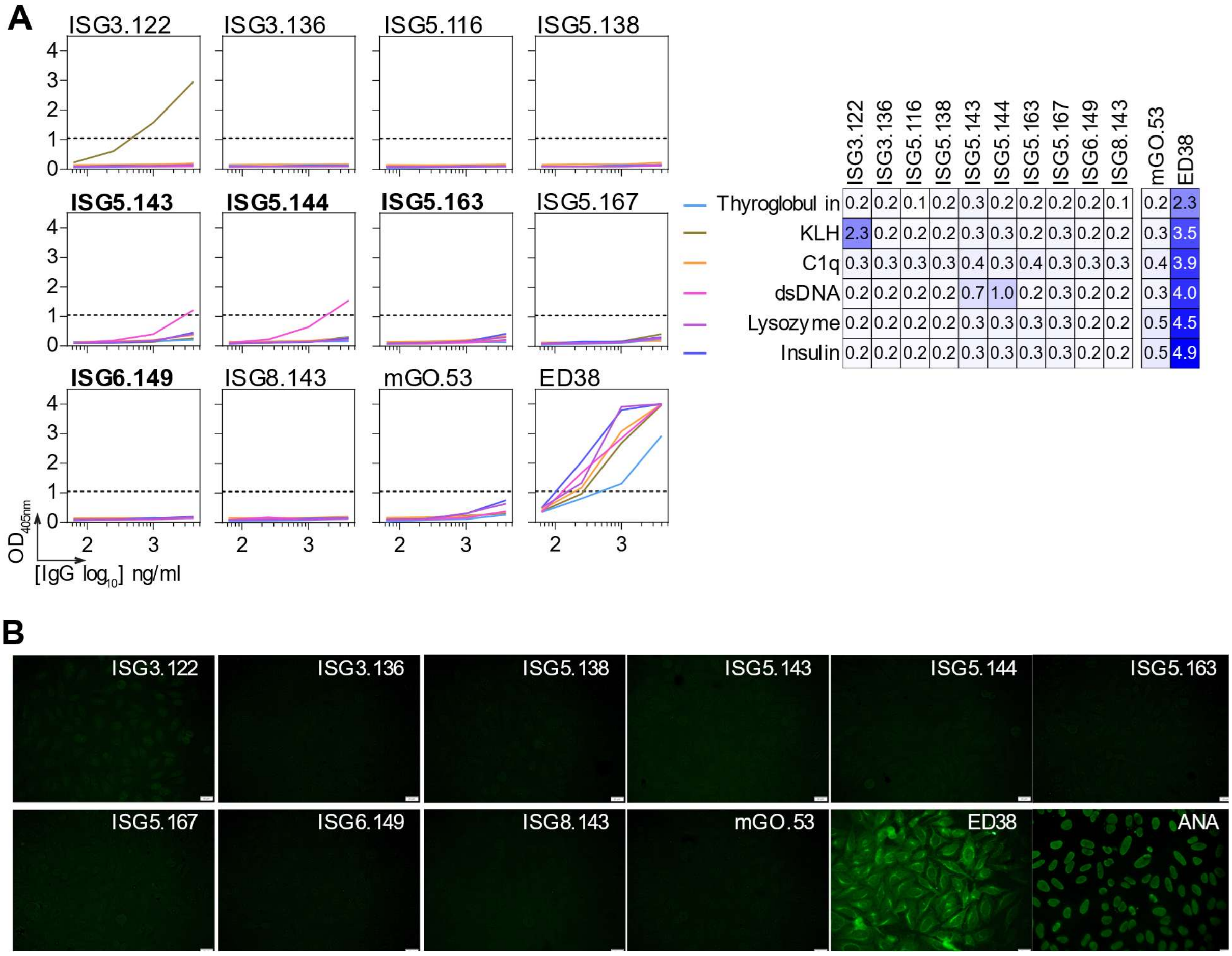

Figure S10. **Off-target binding characteristics of central repeat region (CRR) antibodies**. (**A**) Heatmap (right) showing the ELISA reactivity of selected CRR antibodies against a panel of structurally unrelated antigens. Polyreactive (ED38) and non-polyreactive (mGO.53) controls are included. Color values are proportional to the reactivity level measured as the area under the curve (AUC) of the ELISA graphs (left). (**B**) Microscopic images showing the reactivity of the antibodies in (A) to Hep2-expressing self-antigens assayed by indirect immunofluorescence assay. The negative (mGO.53) and positive (ED38, and kit's ANA) controls are included. The scale bars represent 40 μm.

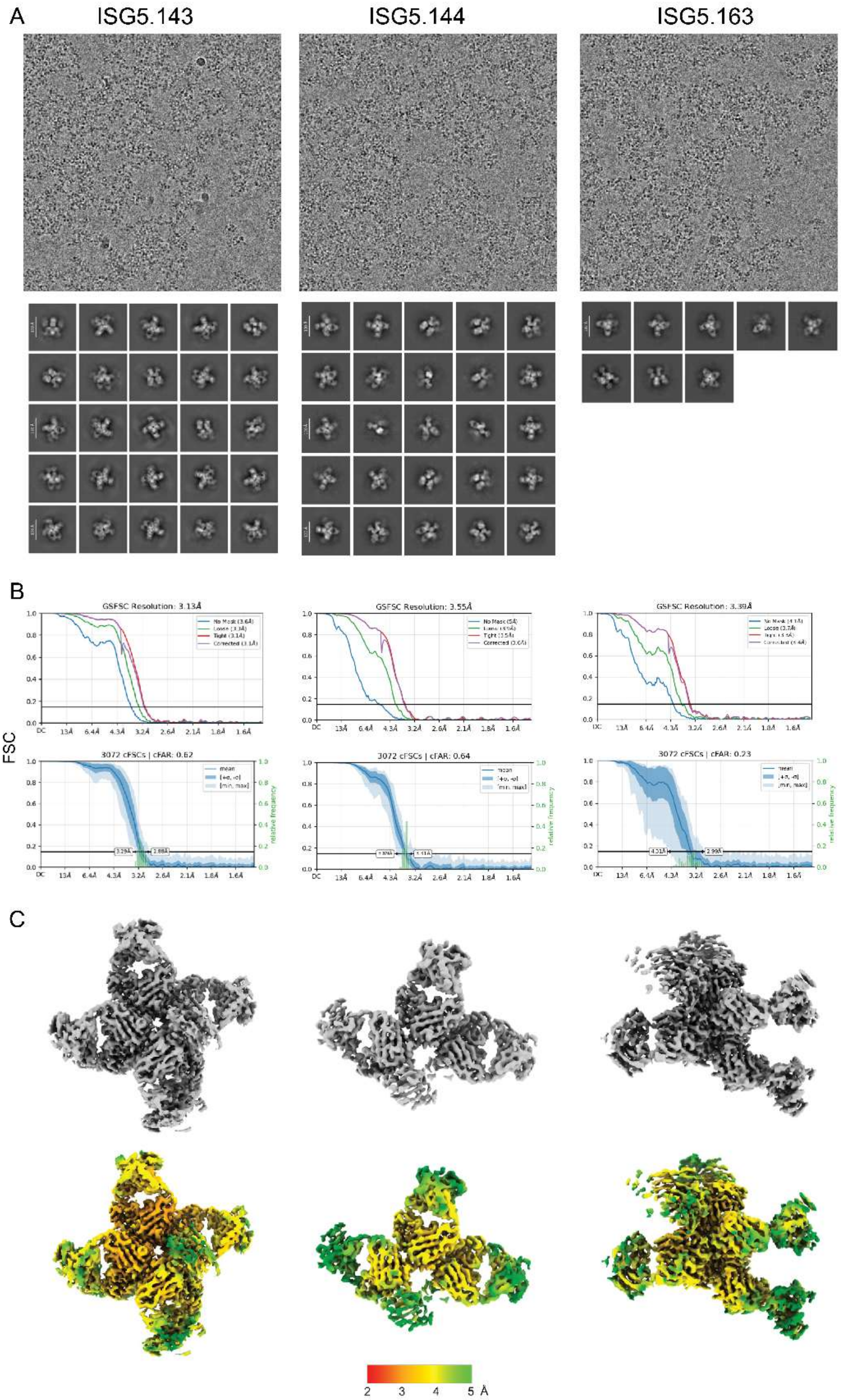

Figure S11. **CryoEM data processing of rsCSP protein in complex with ISG5.143, ISG5.144 and ISG5.163 Fabs**. (**A**) Representative electron micrograph (top), with a 3 Å low-pass filter for better contrast, and 2D classes (bottom). (**B**) Gold-standard Fourier-shell correlation (FSC) and cFAR FSC. (**C**) Overall map 3D volume, uncolored (top) and colored by local resolution (bottom).

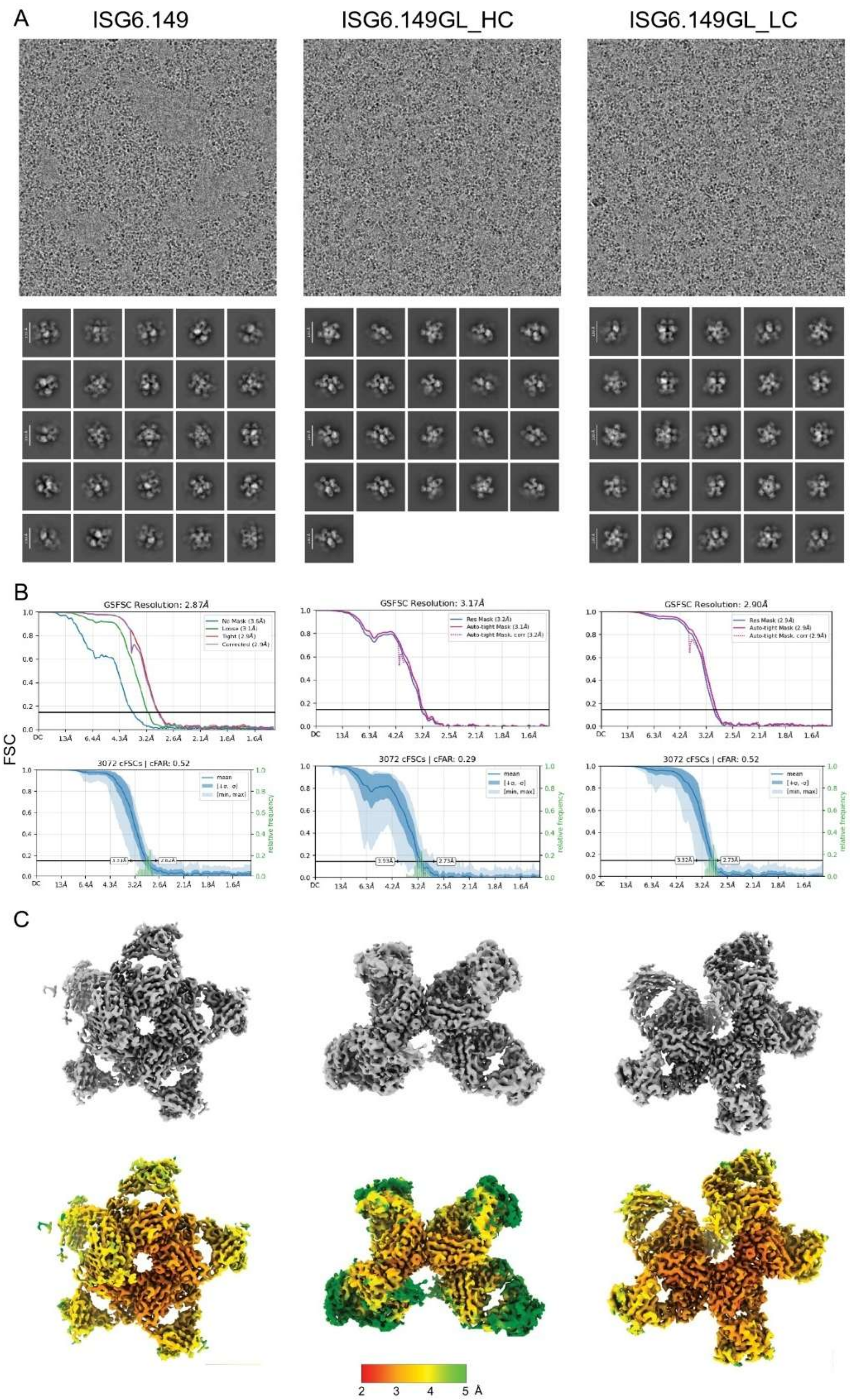

Figure S12. **CryoEM data processing of rsCSP protein in complex with ISG6.149, ISG6.149GL_HC and ISG6.149GL_LC Fabs**. (**A**) Representative electron micrograph (top), with a 3 Å low-pass filter for better contrast, and 2D classes (bottom). (**B**) Gold-standard fourier-shell correlation (FSC) and cFAR FSC. (**C**) Overall map 3D volume, uncolored (top) and colored by local resolution (bottom).

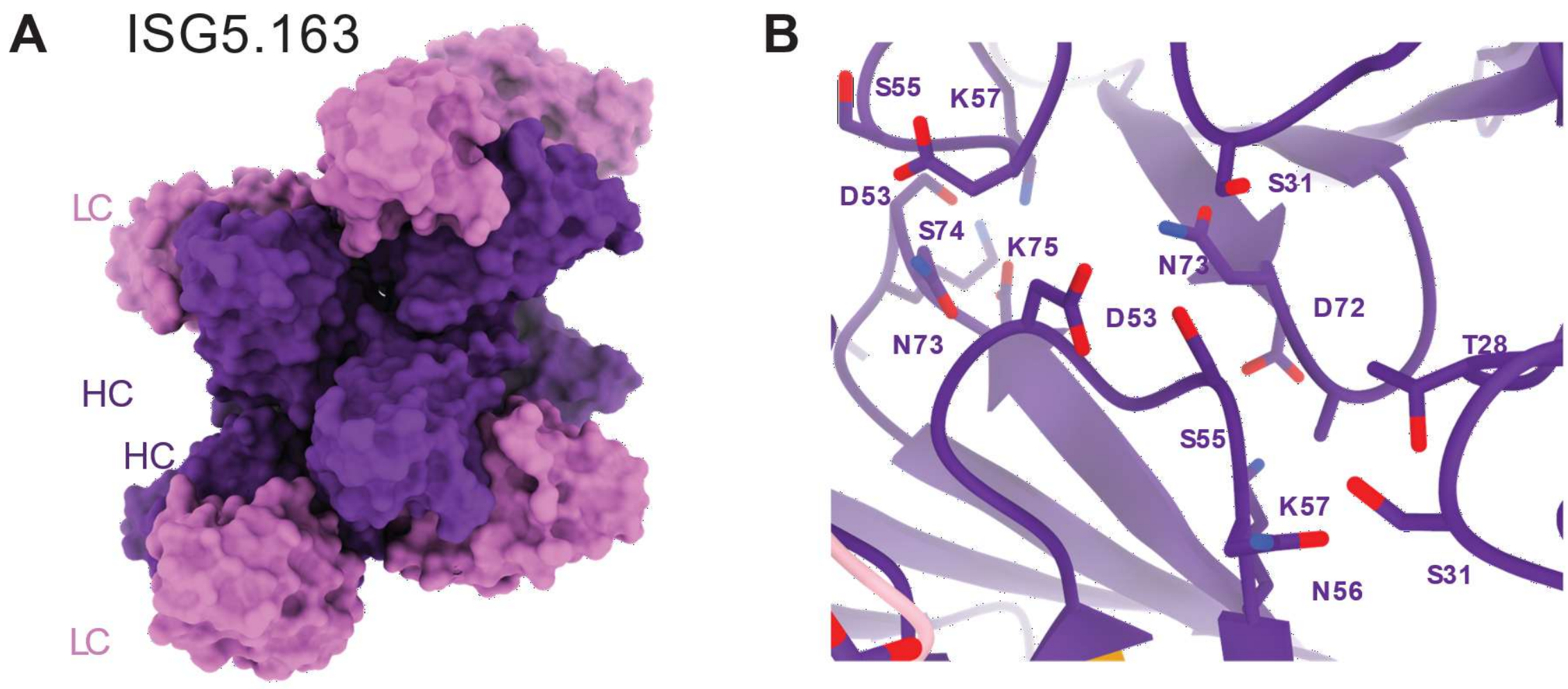

Figure S13. Stacking of ISG5.163 homotypic spirals. (**A**) Surface molecular representation of two spirals of ISG5.163 Fabs, mirroring each other and interacting by the heavy chain (HC). (**B**) Structural details of the interface between HC Fabs. Contacting residues are shown as sticks with heteroatoms colored (blue: N, red: O). Color key: purple (HC) and pink (light chain, LC).

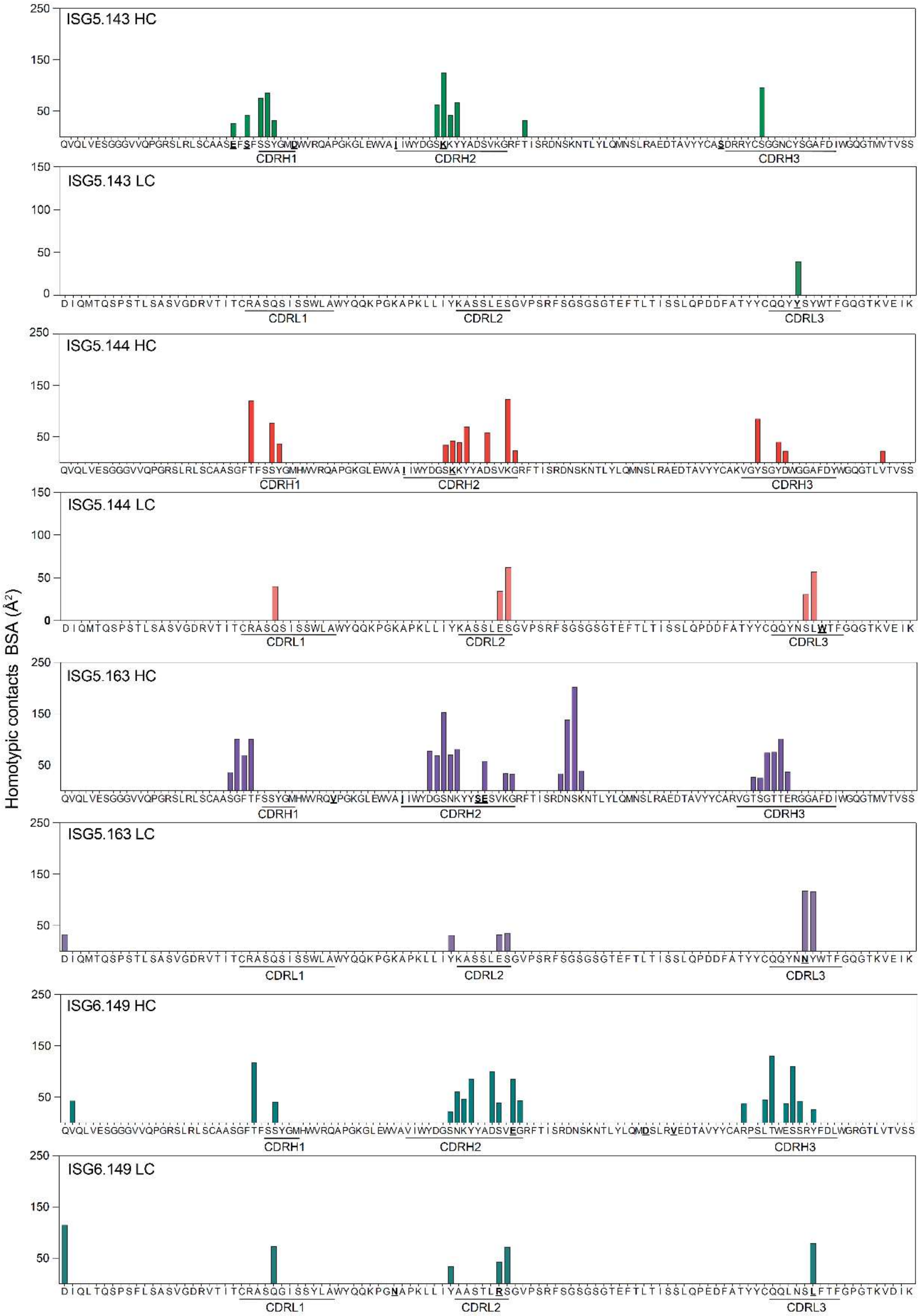

Figure S14. **Homotypic interfaces of ISG5.143, ISG5.144, ISG5.163 and ISG6.149**. Bar graphs showing per residue BSA contributions to Fab-Fab homotypic interfaces with rsCSP. Somatically mutated residues are highlighted in bold and underlined. HC, heavy chain. LC, light chain.

ISG5.143

```
ISG5.143GL_HC  QVQLVESGGGVVQPGRSLRLSCAASGFTFSSYGMHWVRQAPGKGLEWVAVIWYDGSNKYYADS
ISG5.143H      .........................E.S......D..............I......K......

ISG5.143GL_HC  VKGRFTISRDNSKNTLYLQMNSLRAEDTAVYYCARDRRYCSGGNCYSGAFDIWGQGTMVTVSS
ISG5.143H      ..................................S............................

ISG5.143GL_LC  DIQMTQSPSTLSASVGDRVTITCRASQSISSWLAWYQQKPGKAPKLLIYKASSLESGVPSRF
ISG5.143L      ..............................................................

ISG5.143GL_LC  SGSGSGTEFTLTISSLQPDDFATYYCQQYNSYWTFGQGTKVEIK
ISG5.143L      .............................Y..............
```

ISG5.144

```
ISG5.144GL_HC  QVQLVESGGGVVQPGRSLRLSCAASGFTFSSYGMHWVRQAPGKGLEWVAVIWYDGSNKYYAD
ISG5.144H      .................................................I......K.....

ISG5.144GL_HC  SVKGRFTISRDNSKNTLYLQMNSLRAEDTAVYYCAKVGYSGYDWGGAFDYWGQGTLVTVSS
ISG5.144H      .............................................................

ISG5.144GL_LC  DIQMTQSPSTLSASVGDRVTITCRASQSISSWLAWYQQKPGKAPKLLIYKASSLESGVPSRF
ISG5.144L      ..............................................................

ISG5.144GL_LC  SGSGSGTEFTLTISSLQPDDFATYYCQQYNSYWTFGQGTKVEIK
ISG5.144L      ...............................L............
```

ISG5.163

```
ISG5.163GL_HC  QVQLVESGGGVVQPGRSLRLSCAASGFTFSSYGMHWVRQAPGKGLEWVAVIWYDGSNKYYADS
ISG5.163H      .......................................V.........I..........SE.

ISG5.163GL_HC  VKGRFTISRDNSKNTLYLQMNSLRAEDTAVYYCARVGTSGTTERGGAFDIWGQGTMVTVSS
ISG5.163H      .............................................................

ISG5.163GL_LC  DIQMTQSPSTLSASVGDRVTITCRASQSISSWLAWYQQKPGKAPKLLIYKASSLESGVPS
ISG5.163L      ............................................................

ISG5.163GL_LC  RFSGSGSGTEFTLTISSLQPDDFATYYCQQYNSYWTFGQGTKVEIK
ISG5.163L      ................................N.............
```

ISG6.149

```
ISG6.149GL_HC  QVQLVESGGGVVQPGRSLRLSCAASGFTFSSYGMHWVRQAPGKGLEWVAVIWYDGSNKYYAD
ISG6.149H      ..............................................................

ISG6.149GL_HC  SVKGRFTISRDNSKNTLYLQMNSLRAEDTAVYYCARPSLTWESSRYFDLWGRGTLVTVSS
ISG6.149H      ..E..................D...V..................................

ISG6.149GL_LC  DIQLTQSPSFLSASVGDRVTITCRASQGISSYLAWYQQKPGKAPKLLIYAASTLQSGVPSRF
ISG6.149L      .........................................N............R.......

ISG6.149GL_LC  SGSGSGTEFTLTISSLQPEDFATYYCQQLNSYFTFGPGTKVDIK
ISG6.149L      ...............................L............
```

Figure S15. **Germline-reverted variants of ISG5.143, ISG5.144, ISG5.163 and ISG6.149.** Amino acid sequence alignment of the heavy and light chains and inferred germline-reverted variant.

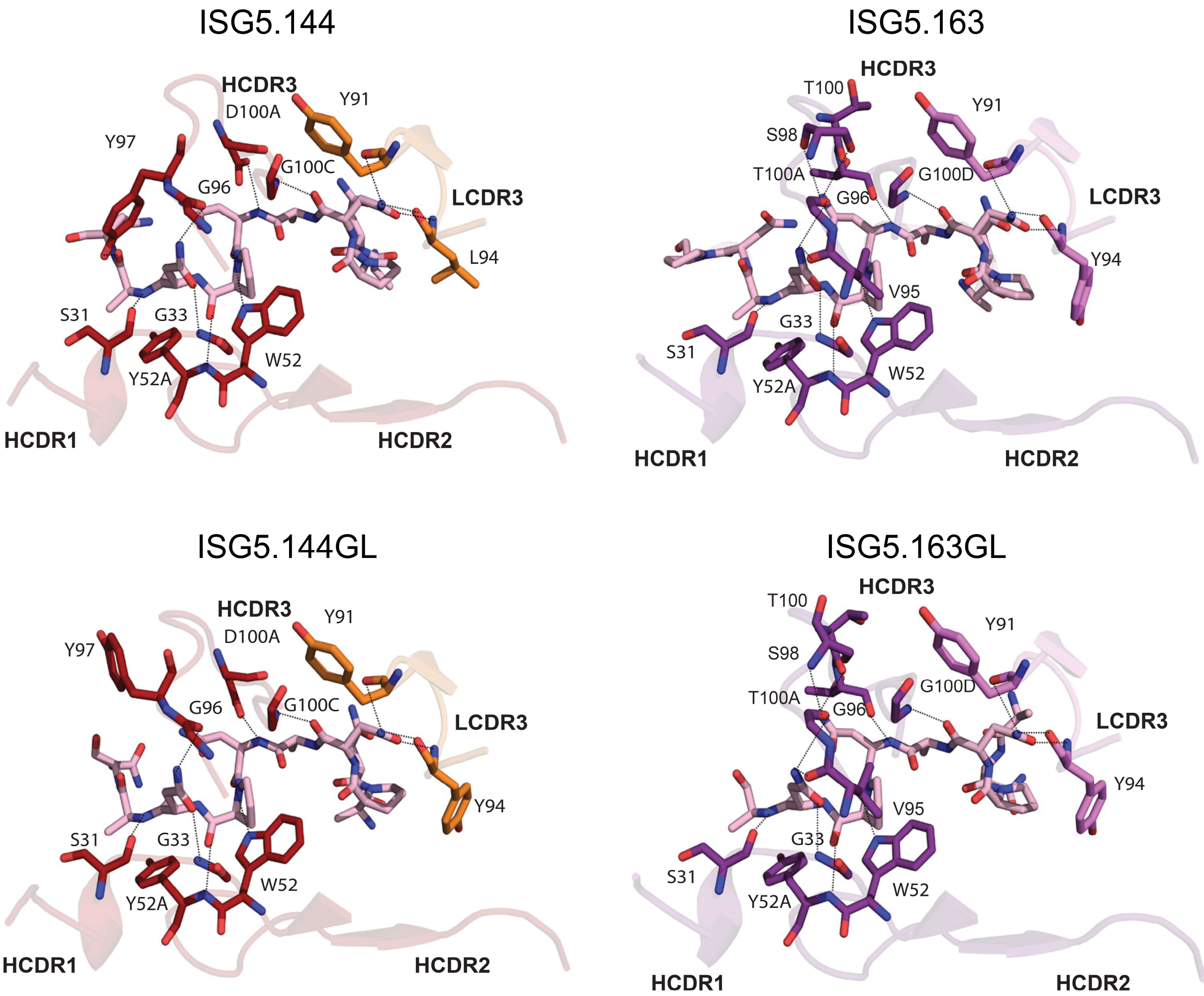

Figure S16. **Co-crystal structures of mutated and germline-reverted variants of ISG5.144 and ISG5.163 Fabs**. Comparison of crystal structures of matured (top) and GL-reverted variants (bottom) Fabs bound to NPNA epitope.

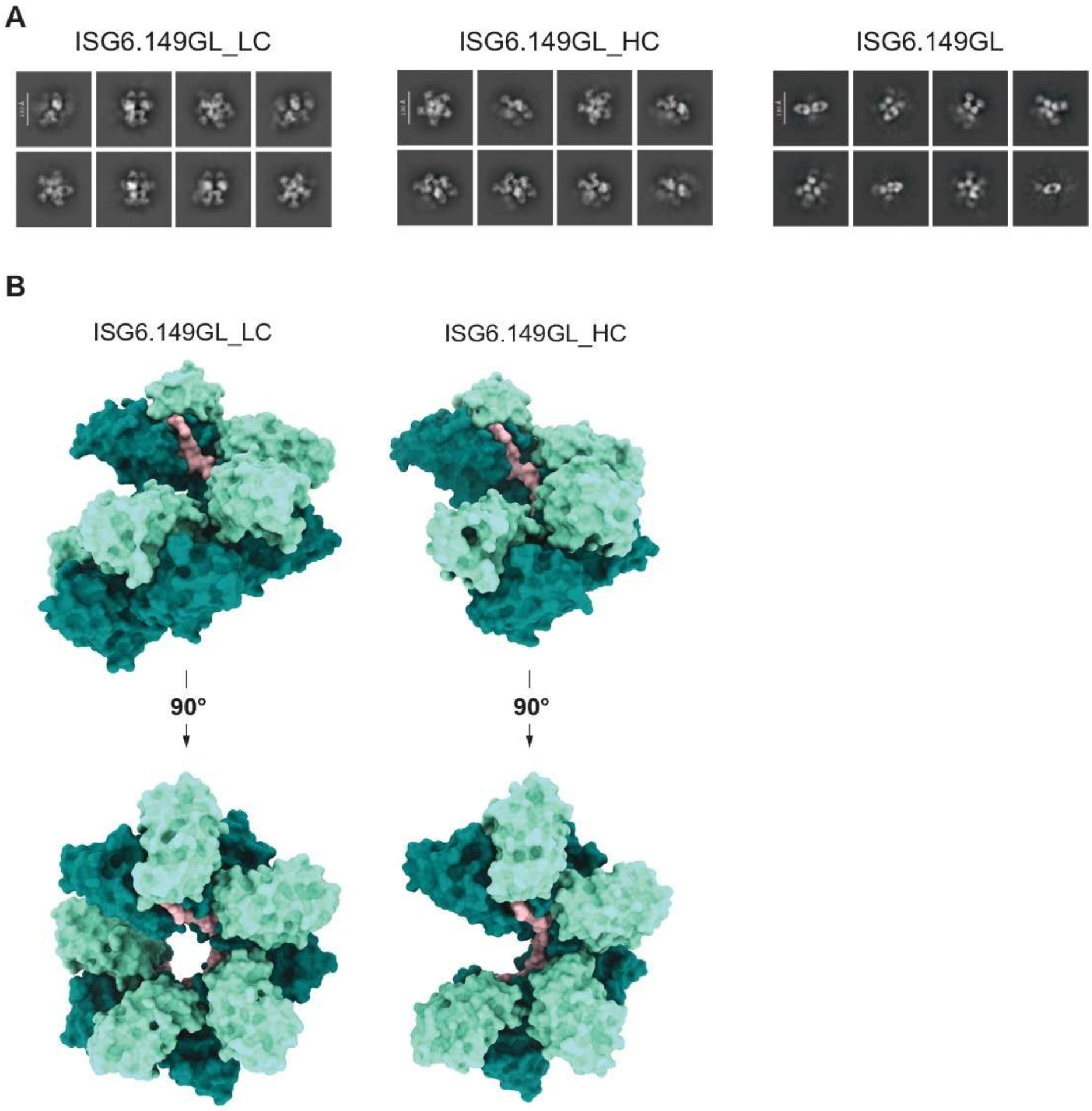

Figure S17. **Cryo-EM analysis of GL-reverted variants of ISG6.149**. (**A**) Representative 2D classes of ISG6.149GL, ISG6.149GL_HC and ISG6.149GL_LC Fabs in complex with rsCSP protein. (**B**) Surface molecular representation of rsCSP (pink) in complex with the indicated Fabs, with heavy and light chains colored in teal and aquamarine, respectively. ISG6.149GL did not form homotypic spirals, and 3D reconstruction was not obtained.

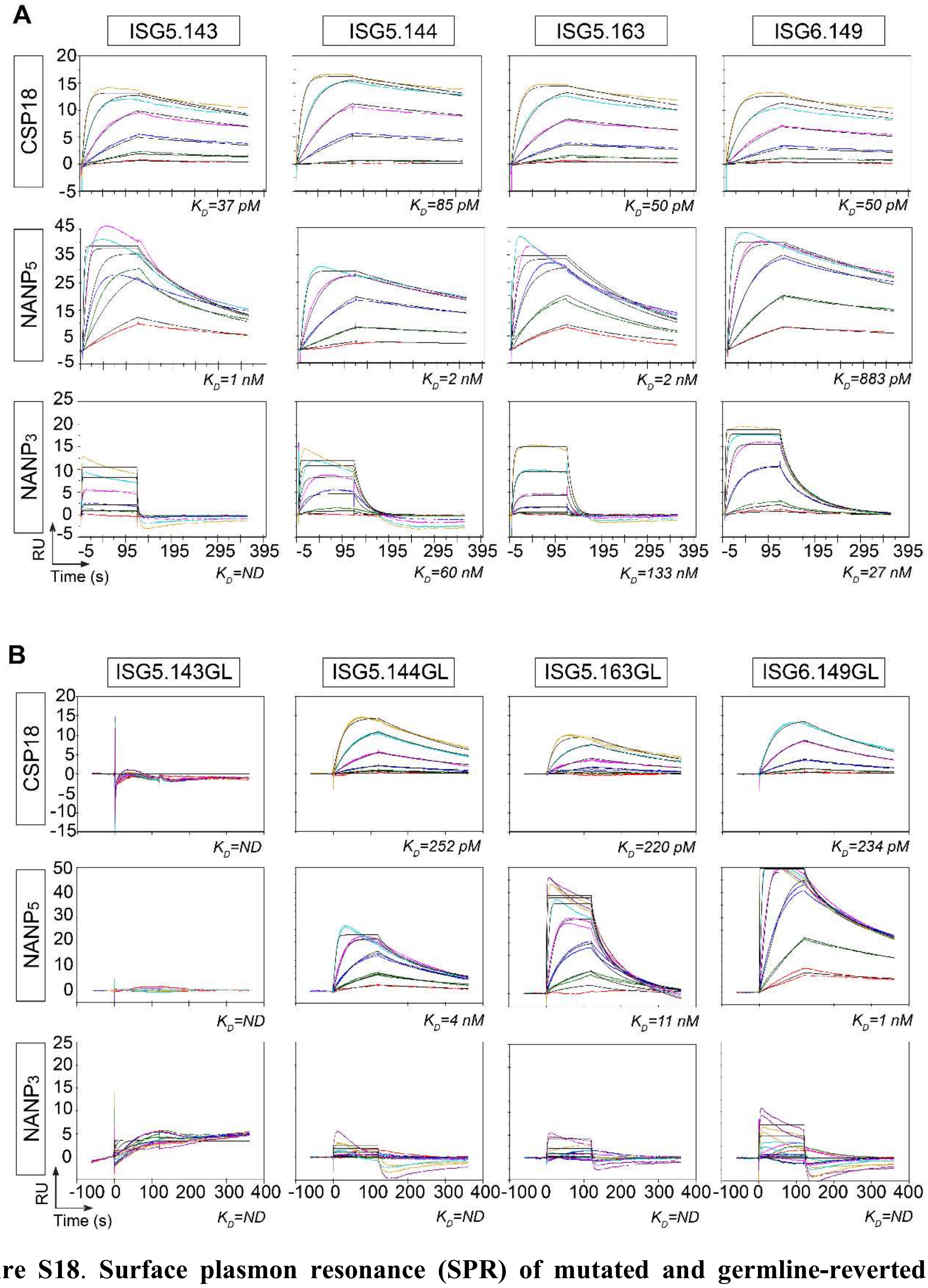

Figure S18. **Surface plasmon resonance (SPR) of mutated and germline-reverted antibodies**. Representative multi-cycle kinetics (MCK) sensorgrams of mutated (**A**) and germline-reverted variants (**B**) of ISG5.143, ISG5.144, ISG5.163 and ISG6.149 antibodies, bound to CSP18, $NANP_5$ and $NANP_3$.

Table S1. Characteristics of RTS,S vaccinated donors

	Age*				CSP18 IgG ELISA (AUC)			
Donor ID	Weeks	Months	Gender	Site	M0	M3	CSP18-reactive IgG^{+} B cells (%)	n CSP18 mAbs
d3	27	6	M	Manhiça	0.4	9.1	1.4	9
d4	9	2	M	Manhiça	0.5	5.8	1.2	13
d5	9	2	F	Manhiça	0.3	7.6	0.95	16
d6	9	2	F	Manhiça	0.4	6.1	0.41	21
d8	38	8	M	Manhiça	0.3	8.2	0.82	10
d9	9	2	F	Manhiça	0.4	7.4	0.84	19

Table S2. Immunoglobulin gene repertoire and reactivity of *Pf*CSP memory B-cell antibodies from RTS,S vaccinated donors

		IgH				CDRH3						IgL			CDRL3							*Pf*CSP reactivity: *Pf*CSP screening (AUC)			*Pf*CSP peptide mapping (AUC)									*In vitro* antiparasitic properties: Lethality		CSPR		Inhibition cell infection (%)		ADCP
mAb	Donor	VH	DH	JH	Subtype	Sequence (aa)	Length	(+)	(-)	VH MUT	SHM (%)	IgL	VL	JL	Sequence (aa)	Length	(+)	(-)	VL MUT	SHM (%)	Clonotype members (N)	CSP18	NANP10	CTD	Jt1	Jt2	M1	M2	NANP3	NANP4	NANP5	NANP6	NANP10	viability 100 µg/ml (%)	EC50 (µg/ml)	100 µg/ml	10 µg/ml	3 µg/ml	1 µg/ml	score
ISG3.101	d3	3-33*01	3-22*01	4*02	γ1	AIDNYDRSDYSTFDY	15	1	4	2	0,7	κ	1-5*03	1*01	QQYNSYWT	8	0	0	6	2,3	1	5,7	5,7	0,6	0,3215	6,411	0,578	12,3	10,74	11,72	12,88	13,14	13,2	9,0	8,2	1712	1659	74,2	29,9	9,5
ISG3.102	d3	3-33*06	3-9*01	6*03	γ1	DHDYFAPNYMDV	12	1	3	8	2,7	κ	3-11*01	2*01	QQRSNWPPFT	10	1	0	7	2,6	1	5,7	5,5	0,2	0,3433	0,272	0,2852	0,2947	0,3294	0,2768	0,3948	3,237	9,2	50,6	100	1365	1249	16,7	8,0	1,1
ISG3.103	d3	3-21*01	3-9*01	3*02	γ1	DFGTELRHFDWSDALDI	17	2	5	7	2,4	κ	3-20*01	4*01	QQYGSSLT	8	0	0	4	1,5	1	5,7	0,2	5,7	ND	ND	ND	ND	ND	ND	ND	ND	ND	75,9	ND	1295	ND	17,7	ND	ND
ISG3.109	d3	3-23*04	7-27*01	3*02	γ1	NTLSGNWGPDAFDI	14	0	2	13	4,5	κ	3-11*01	4*01	QQRSRRPT	8	3	0	8	3,0	1	5,7	0,1	5,7	ND	ND	ND	ND	ND	ND	ND	ND	ND	82,4	ND	1268	ND	1,3	ND	ND
ISG3.114	d3	3-21*01	3-3*01	6*03	γ1	GDSFWSGYSNSRVYYSCMDV	20	1	2	3	1,0	κ	3-20*01	1*01	QQYGSSPPWT	10	0	0	7	2,6	1	5,7	0,1	5,4	ND	ND	ND	ND	ND	ND	ND	ND	ND	80,6	ND	1293	ND	3,1	ND	ND
ISG3.120	d3	7-4-1*02	4-23*01	2*01	γ1	DYGGNSEDWYFDL	13	0	4	9	3,0	λ	3-21*03	1*01	QVWDSSSDHGV	11	1	2	8	2,8	1	5,7	0,1	5,6	ND	ND	ND	ND	ND	ND	ND	ND	ND	80,1	ND	1195	ND	18,4	ND	ND
ISG3.122	d3	3-33*06	6-19*01	4*02	γ1	DGRGLSGVAGTGFFDF	16	1	2	5	1,7	λ	1-40*01	1*01	QSYDSSLGAFV	11	0	1	5	1,7	1	5,7	5,7	1,4	0,3397	3,99	6,145	12,99	12,13	12,25	13,11	13,11	13,1	11,9	6,5	1568	1428	92,2	33,1	15,2
ISG3.126	d3	3-21*01	7-27*01	5*02	γ1	DPSSGNWGAWFDP	13	0	2	6	2,0	λ	3-21*02	2*01	QVWDTTSDHPGV	12	1	2	9	3,2	1	5,7	0,1	5,7	ND	ND	ND	ND	ND	ND	ND	ND	ND	83,3	ND	1316	ND	0	ND	ND
ISG3.136	d3	3-33*06	1-7*01	4*02	γ1	VGEGTAERGGAFDY	14	1	3	9	3,1	κ	1-5*03	1*01	QQYNSLWT	8	0	0	10	3,8	1	5,7	5,7	0,5	0,3448	8,144	10,61	13,12	12,57	12,01	13,2	13,24	13,1	10,7	5,8	1494	1554	80,0	17,0	14,6
ISG4.103	d4	4-38-2*01	3-22*01	5*02	γ1	AITMVIVVITELHNWFDP	18	1	2	10	3,5	κ	3-15*01	5*01	QQYNSLWT	9	0	0	2	1,9	1	5,7	0,3	5,7	ND	ND	ND	ND	ND	ND	ND	ND	ND	71,9	ND	1283	ND	19,1	ND	ND
ISG4.106	d4	3-11*04	3-22*01	4*02	γ1	GRTNMMVHDTYFDY	14	2	2	13	4,4	λ	3-1*01	2*01	QAWDSSTYVV	10	0	1	6	2,2	1	5,7	0,2	5,7	ND	ND	ND	ND	ND	ND	ND	ND	ND	81,4	ND	1230	ND	7,2	ND	ND
ISG4.107	d4	3-30*02	6-19*01	4*02	γ1	DGGWYYFDY	9	0	2	8	2,7	κ	4-1*01	2*03	QQCYSSPYS	9	0	0	6	2,1	1	3,8	3,7	0,2	0,3373	0,2696	0,2741	0,3062	0,3138	0,2827	0,2911	2,254	4,5	77,3	100	1265	1261	0	12,1	1,1
ISG4.109	d4	1-69*18	1-7*01	4*02	γ1	GRRGWNFPRYFDY	13	3	1	10	3,4	κ	1-33*01	5*01	QQYDNLPSIT	10	0	1	3	1,1	5	5,7	0,4	5,7	ND	ND	ND	ND	ND	ND	ND	ND	ND	83,0	ND	1270	ND	21,6	ND	ND
ISG4.110	d4	3-30*02	7-27*01	3*02	γ1	NWVEALDI	8	0	2	17	5,8	κ	1-39*01	2*01	QQSYSSRPYT	10	1	0	6	2,3	1	5,7	0,2	5,7	ND	ND	ND	ND	ND	ND	ND	ND	ND	78,0	ND	1226	ND	0	ND	ND
ISG4.119	d4	3-64D*09	5-18*01	5*02	γ1	SATDSFSWYNWFDP	14	0	2	7	2,4	λ	1-44*01	3*02	AAWDDSLNGWV	11	0	2	2	0,7	1	5,7	0,2	5,7	ND	ND	ND	ND	ND	ND	ND	ND	ND	74,1	ND	1261	ND	12,4	ND	ND
ISG4.124	d4	3-23*04	5-18*01	4*02	γ1	GRGYSYGVFDY	13	1	1	10	3,4	κ	1-33*01	2*01	QQYDNLPYT	9	0	1	5	1,9	1	5,7	0,1	5,7	ND	ND	ND	ND	ND	ND	ND	ND	ND	75,6	ND	1290	ND	0	ND	ND
ISG4.128	d4	3-23*04	5-18*01	4*02	γ1	GLGYSYGRFDS	13	1	1	20	6,8	κ	1-33*01	2*01	QQYDNLPYT	9	0	1	5	1,9	1	5,7	0,1	5,7	ND	ND	ND	ND	ND	ND	ND	ND	ND	81,1	ND	1306	ND	11,6	ND	ND
ISG4.129	d4	5-10-1*01	6-13*01	5*02	γ1	HISSSSWVHSSWFDP	15	2	1	4	1,4	κ	3-15*01	2*01	QQYNNWPPEMYT	12	0	1	2	0,7	1	5,7	0,2	5,1	ND	ND	ND	ND	ND	ND	ND	ND	ND	81,4	ND	1307	ND	3,1	ND	ND
ISG4.135	d4	3-30*02	6-19*01	4*02	γ1	VGDGTVAGHFDY	12	1	2	12	4,1	κ	2-30*01	2*01	MQGTHWPPT	9	1	0	1	0,4	1	5,7	5,7	0,2	0,3232	0,3209	0,2763	7,818	0,405	4,183	8,403	12,08	13,1	11,8	23,1	1588	1337	68,9	40,1	16,9
ISG4.157	d4	3-64D*09	1-1*01	6*02	γ1	GELERRGYYYNGMDV	15	2	3	0	0,0	λ	3-1*01	2*01	QAWDSSTLGVV	11	0	1	4	1,4	1	5,7	0,1	5,7	ND	ND	ND	ND	ND	ND	ND	ND	ND	82,0	ND	1229	ND	20,6	ND	ND
ISG4.171	d4	1-2*06	6-19*01	5*02	γ1	DVWGGSSGYPFDP	13	0	2	4	1,4	κ	2D-29*01	1*01	MQSIQLPRT	9	1	0	9	3,2	1	5,7	5,7	0,2	0,3106	0,2827	0,2661	2,366	0,3167	0,4153	7,838	11,67	13,1	10,3	6,0	1480	1583	96,7	23,9	3,2
ISG5.108	d5	3-21*01	1-7*01	4*02	γ3	PSIMFNWNYYFDY	13	0	1	4	1,4	κ	3-15*02	5*01	QQYDNWPPIT	10	0	1	4	1,5	1	5,7	0,3	5,7	ND	ND	ND	ND	ND	ND	ND	ND	ND	74,8	ND	1275	ND	0,5	ND	ND
ISG5.110	d5	3-33*01	3/OR15-3a*01	3*02	γ3	VRSGPANLVRDWTGAFDI	18	2	2	5	1,7	κ	1-5*03	1*01	QQYNSYWT	8	0	0	7	2,6	1	5,7	5,7	0,2	0,3364	1,696	2,92	12,2	11,64	13,13	13,22	13,25	13,3	10,3	13,8	1513	1381	42,4	20,6	13,3
ISG5.112	d5	3-21*01	3-3*01	4*02	γ1	AGNFWSGPYDY	11	0	1	0	0,0	κ	3-15*02	1*01	QQYNNWPWT	9	0	0	2	0,8	1	1,9	0,1	3,4	ND	ND	ND	ND	ND	ND	ND	ND	ND	ND	ND	ND	ND	ND	ND	ND
ISG5.116	d5	3-33*06	2-2*01	3*02	γ1	VGDGGFCSGTYCHGDAFDI	19	1	3	3	1,0	κ	3-15*02	1*01	QQYNNWWT	8	0	0	3	1,1	1	5,6	5,7	0,2	0,3279	0,4664	0,8733	9,045	7,09	12,68	13,11	13,21	13,3	9,7	7,2	1590	1587	80,3	28,8	13,8
ISG5.118	d5	3-73*02	2-15*01	5*02	γ1	AVVGQDWFGP	10	0	1	12	4,0	λ	3-9*01	2*01	QVWDSSTHVV	10	1	1	5	1,8	1	5,7	0,1	5,7	ND	ND	ND	ND	ND	ND	ND	ND	ND	82,2	ND	1254	ND	0	ND	ND
ISG5.130	d5	3-33*06	2-2*01	3*01	γ1	VGDGGYCSSTSCHGDAFDL	19	1	3	0	0,0	κ	3-15*02	1*01	QQYNNWWT	8	0	0	7	2,7	3	5,7	5,7	0,2	0,3381	1,053	1,915	11,02	6,699	11,89	12,98	13,12	13,2	9,2	11,8	1651	1590	60,6	20,6	14,1
ISG5.138	d5	3-33*01	1-7*01	3*02	γ1	VCNEACQGKGTTSLAFDI	18	1	2	5	1,7	κ	3-15*02	1*01	QQYNNWWT	8	0	0	6	2,3	1	5,7	5,7	0,2	0,3343	10,14	9,981	12,68	12,37	13,12	13,22	13,26	13,1	7,5	6,3	1630	1469	77,6	12,9	9,8
ISG5.143	d5	3-33*01	2-15*01	3*02	γ1	DRRYCSGGNCYSGAFDI	17	2	2	9	3,0	κ	1-5*03	1*01	QQYYSYWT	8	0	0	1	0,4	1	5,7	5,7	0,2	0,3254	0,2956	0,2751	6,23	6,241	12,61	13,08	13,17	13,3	9,6	6,5	1741	1667	91,2	32,9	11,2
ISG5.144	d5	3-33*06	5-12*01	4*02	γ1	VGYSGYDWGGAFDY	14	0	2	2	0,7	κ	1-5*03	1*01	QQYNSLWT	8	0	0	6	2,3	1	5,7	5,7	0,2	0,3369	5,98	6,196	12,74	11,83	12,54	13,15	13,1	13,2	9,3	12,0	1530	1562	68,7	7,7	12,7
ISG5.145	d5	3-21*01	6-19*01	4*02	γ1	RWGIAVRYFDY	11	2	1	8	2,7	λ	3-21*03	2*01	QVWDSSSDHPDVV	13	1	3	4	1,4	1	5,7	0,2	5,7	ND	ND	ND	ND	ND	ND	ND	ND	ND	77,1	ND	1273	ND	0	ND	ND
ISG5.147	d5	3-33*06	2-15*01	4*02	γ1	GGQWGSGEFDY	11	0	2	0	0,0	κ	4-1*01	1*01	QQYYSTPWT	9	0	0	1	0,4	1	3,6	2,3	0,2	0,3369	0,2553	0,3479	0,2916	0,3029	0,4069	0,5419	0,5957	3,8	79,4	100	1310	1263	0	3,4	1,3
ISG5.154	d5	3-33*06	6-13*01	4*02	γ1	VGSSSWYYFDY	11	0	1	0	0,0	λ	6-57*01	3*02	QSYDWV	6	0	1	0	0,0	1	5,7	5,7	0,2	0,3197	0,2588	0,2846	0,2854	0,3158	0,3197	4,453	6,71	10,9	78,6	100	1286	1260	0	13,3	1,0
ISG5.160	d5	3-21*01	2-2*01	3*02	γ1	DRGRYCSTTVCYDAFDI	17	2	3	6	2,0	κ	3-20*01	2*01	QQYGSSRYTT	10	1	0	4	1,5	1	5,7	0,1	5,7	ND	ND	ND	ND	ND	ND	ND	ND	ND	82,1	ND	1256	ND	0	ND	ND
ISG5.163	d5	3-33*01	1-1*01	3*02	γ1	VGTSGTTERGGAFDI	15	1	2	4	1,4	κ	1-5*03	1*01	QQYNNYWT	8	0	0	2	0,8	1	5,7	5,7	0,2	0,3269	10,61	12,29	13,01	12,9	12,36	13	12,99	13,4	11,5	8,0	1680	1700	80,4	20,1	8,9
ISG5.166	d5	3-7*01	N/A	4*02	γ1	YLNY	4	0	0	4	1,4	κ	2D-29*02	5*01	MQSIQLPIT	9	0	0	2	0,7	1	5,7	5,7	0,2	12,57	0,2839	0,3118	10,62	10	12,04	13,12	13,12	13,3	12,0	16,0	1302	1302	22,8	33,6	2,6
ISG5.167	d5	3-33*01	4-17*01	3*02	γ1	VQADNDYVAFDI	12	0	3	11	3,7	κ	1-5*03	1*01	QQYNSYWT	8	0	0	3	1,2	2	5,7	5,7	0,2	0,3112	0,2743	0,3052	11,17	11,29	11,62	12,97	13,04	13,2	12,2	5,9	1400	1641	91,5	23,7	8,2
ISG5.170	d5	4-39*07	6-19*01	1*01	γ1	EEREILAGTVMFSRYFQH	18	3	3	12	4,0	λ	1-47*01	2*01	AAWDDSLSGVV	11	0	2	3	1,0	1	5,7	0,1	5,7	ND	ND	ND	ND	ND	ND	ND	ND	ND	78,3	ND	1316	ND	14,9	ND	ND
ISG5.174	d5	3-48*01	6-13*01	4*02	γ1	LGLIAAAGTRYFDY	14	1	1	4	1,4	λ	3-21*01	2*01	QVWDSGSDHPV	11	1	2	10	3,5	1	5,7	0,2	5,7	ND	ND	ND	ND	ND	ND	ND	ND	ND	82,8	ND	1304	ND	15,1	ND	ND
ISG5.179	d5	3-33*06	3-22*01	3*02	γ1	VGSSESSGYYKPTEGWTDAFDI	22	1	4	2	0,7	κ	1-5*03	1*01	QQYNFLWT	8	0	0	2	0,8	1	5,7	5,7	0,2	0,3313	3,29	1,003	10,88	10,9	13,13	13,27	13,29	13,3	9,7	17,9	1562	1399	52,0	9,2	5,3
ISG5.184	d5	3-48*01	6-13*01	4*02	γ1	LGSSWTYYFDY	11	0	1	8	2,7	λ	3-21*03	2*01	QVWDTTSDLVV	11	0	2	5	1,8	1	5,7	0,2	5,7	ND	ND	ND	ND	ND	ND	ND	ND	ND	81,2	ND	1200	ND	17,5	ND	ND
ISG6.102	d6	3-21*01	3-16*01	4*02	γ1	DDLGVRGLGDYFDY	14	1	4	7	2,4	λ	3-1*01	1*01	QAWDSSTYV	9	0	1	9	3,2	1	5,7	0,1	5,7	ND	ND	ND	ND	ND	ND	ND	ND	ND	81,7	ND	1369	ND	5,0	ND	ND
ISG6.104	d6	7-4-1*02	1-26*01	6*02	γ1	VRWELLGYYYGMDV	14	1	2	1	0,3	κ	4-1*01	4*01	QQYYITPPLT	10	0	0	2	0,7	1	5,7	5,7	0,2	0,3279	12,78	4,18	13,25	8,83	12,91	13,25	13,25	13,3	9,1	29,5	1456	1334	26,3	18,4	7,5
ISG6.106	d6	3-30*01	2-2*01	4*02	γ1	GCSTSCVLGDRFDY	14	1	2	0	0,0	κ	3-11*01	1*01	QQHSNWPRT	9	2	0	3	1,1	1	5,7	5,7	0,2	0,3276	10,91	9,425	12,99	4,367	11,13	12,91	13,02	13,2	9,5	19,3	1360	1291	18,7	11,1	1,8
ISG6.111	d6	3-48*04	3-3*01	4*02	γ1	RIFGVVQYYFDY	12	1	1	4	1,4	λ	3-1*01	1*01	QAWDSRTAYV	10	1	1	5	1,8	1	5,7	0,1	5,7	ND	ND	ND	ND	ND	ND	ND	ND	ND	83,9	ND	1297	ND	4,7	ND	ND
ISG6.118	d6	3-33*06	3-22*01	5*02	γ2	DLRYFYDISDTGGFDP	16	1	4	5	1,7	λ	1-40*01	1*01	QSYDSRLRGYV	11	2	1	4	1,3	1	5,7	5,7	0,7	0,4672	11	12,38	13,28	11,61	13,3	13,32	13,32	13,1	10,2	6,9	1575	1449	94,7	22,3	13,8
ISG6.139	d6	3-33*06	3/OR15-3a*01	4*02	γ1	SASVFGHGYYFDY	13	1	1	8	2,7	κ	3-11*01	1*01	QQRNNWPPT	9	1	0	5	1,9	1	5,7	5,7	0,2	0,3275	0,3352	0,4301	11,61	5,59	10,38	12,89	12,96	13,1	12,1	24,3	1308	1276	5,8	12,1	1,2
ISG6.141	d6	3-21*01	4-11*01	5*02	γ3	RPGVTTTDNWFDP	13	1	2	12	4,1	λ	3-21*03	1*01	QVWDSSSDYV	10	0	2	2	0,7	1	5,7	0,1	5,7	ND	ND	ND	ND	ND	ND	ND	ND	ND	82,9	ND	1296	ND	3,4	ND	ND
ISG6.149	d6	3-33*01	1-26*01	2*01	γ1	PSLTWESSRYFDL	13	1	2	4	1,4	κ	1-9*01	3*01	QQLNSLFT	8	0	0	4	1,6	2	5,7	5,7	0,2	0,3263	0,2827	0,315	11,6	10,3	10,78	12,92	13,06	13,2	11,4	5,3	1755	1402	92,5	43,7	9,9
ISG6.152	d6	3-21*01	4-23*01	2*01	γ1	AGGLRWIDHWYFDL	14	2	2	5	1,7	λ	1-51*02	3*02	GTWDSSLSAGWV	12	0	1	4	1,4	1	1,2	0,1	2,2	ND	ND	ND	ND	ND	ND	ND	ND	ND	ND	ND	ND	ND	ND	ND	ND
ISG6.158	d6	3-21*01	3-3*01	4*02	γ1	VSGVVIRHFDY	11	2	1	6	2,0	λ	3-21*03	2*01	QVWDSSSDHHVV	12	2	2	4	1,4	1	5,7	0,1	5,7	ND	ND	ND	ND	ND	ND	ND	ND	ND	78,6	ND	1305	ND	10,3	ND	ND
ISG6.160	d6	3-33*01	N/A	4*02	γ1	PIGGHYFDY	9	1	1	1	0,3	κ	2-29*03	1*01	MQGIHLWT	8	1	0	5	1,8	1	5,7	5,7	0,2	0,3278	1,635	9,523	13,02	7,331	12,1	13,15	13,21	13,0	8,3	11,6	1310	1229	37,3	22,2	1,2
ISG6.163	d6	3-48*04	1-7*01	4*02	γ1	DGLYKWNYYFDY	12	1	2	6	2,0	λ	3-21*03	3*02	QVWDSSSDHRV	11	2	2	1	0,4	1	5,7	0,1	5,7	ND	ND	ND	ND	ND	ND	ND	ND	ND	77,0	ND	1309	ND	0	ND	ND
ISG6.165	d6	3-33*01	6-6*01	4*02	γ1	GVWAYSSASDY	11	0	1	4	1,4	κ	1-9*01	2*01	QQLNSYPYT	9	0	0	6	2,2	1	5,5	0,1	5,6	ND	ND	ND	ND	ND	ND	ND	ND	ND	82,4	ND	1241	ND	11,9	ND	ND
ISG6.166	d6	1-8*01	3-3*01	6*02	γ1	GYDFWSGYSRSRNYYYVMDV	20	2	2	1	0,3	κ	3-20*01	5*01	QQYGSSPPEIT	11	0	1	2	0,7	1	5,7	0,1	5,3	ND	ND	ND	ND	ND	ND	ND	ND	ND	81,2	ND	1306	ND	10,6	ND	ND
ISG6.167	d6	3-33*01	1-26*01	3*02	γ1	DRGRGSHGGAFDI	13	3	2	5	1,7	κ	3-11*01	1*01	QQHSNWWT	8	1	0	5	1,9	2	5,7	5,7	0,2	0,397	5,742	12,16	13,16	11,91	12,71	13,24	13,27	13,3	10,3	20,6	1323	1460	14,4	1,0	1,8
ISG6.169	d6	3-21*01	2-21*02	4*02	γ1	GGGGSDSGSYYFDY	14	0	2	6	2,0	κ	3-11*01	4*01	QQRSNWPPLT	10	1	0	2	0,8	1	5,7	0,1	5,7	ND	ND	ND	ND	ND	ND	ND	ND	ND	84,0	ND	1288	ND	9,7	ND	ND
ISG6.170	d6	3-33*01	6-13*01	3*02	γ1	VRNGYSSSWGDAFDI	15	1	2	0	0,0	κ	1-5*03	1*01	QQYNSLWT	8	0	0	4	1,5	1	5,7	5,7	0,2	0,3212	0,2672	0,2686	1,144	5,448	8,209	11,24	11,98	13,0	24,9	74,4	1305	1262	16,4	0	1,7
ISG6.181	d6	3-21*01	6-19*01	3*02	γ3	VSGGRGWFGAFDI	13	1	1	7	2,4	λ	3-21*03	3*02	QVWDNSDHPV	10	1	2	9	3,2	1	5,7	0,1	5,7	ND	ND	ND	ND	ND	ND	ND	ND	ND	82,1	ND	1349	ND	15,8	ND	ND
ISG6.188	d6	3-21*01	6-19*01	4*02	γ1	GWDRGWQRGVDY	12	2	2	6	2,0	λ	1-51*01	2*01	GTWDSSLSAVV	11	0	1	5	1,7	1	5,7	0,1	5,7	ND	ND	ND	ND	ND	ND	ND	ND	ND	81,0	ND	1245	ND	17,5	ND	ND
ISG6.193	d6	3-15*01	1-26*01	5*02	γ3	DYVYSGSYYWFDP	13	0	2	0	0,0	κ	2-28*01	2*01	MQALQTPHT	9	1	0	0	0,0	1	3,2	1,7	0,2	0,3235	0,2911	0,2863	0,3549	0,302	0,4027	0,3495	0,4427	7,9	61,6	100	1276	1262	15,1	2,0	1,2
ISG6.195	d6	3-48*04	4-23*01	4*02	γ1	ATSTVVTLDLIDY	13	0	2	10	3,4	κ	1-5*03	1*01	QHYNSYPWT	9	1	0	2	0,8	1	3,8	0,1	4,6	ND	ND	ND	ND	ND	ND	ND	ND	ND	80,8	ND	1326	ND	9,2	ND	ND
ISG6.196	d6	3-21*01	1-1*01	4*02	γ3	VGGPWTYYFDY	11	0	1	5	1,7	λ	3-21*03	2*01	QVWDSSSNI	9	0	1	4	1,4	1	5,7	0,1	5,7	ND	ND	ND	ND	ND	ND	ND	ND	ND	75,7	ND	1281	ND	6,6	ND	ND
ISG8.106	d8	4-39*01	1-26*01	3*02	γ1	HIGGSHGAAFDI	12	2	1	4	1,3	λ	1-33*01	2*01	QQFDNLPYT	9	0	1	5	2,3	1	4,4	0,1	5,4	ND	ND	ND	ND	ND	ND	ND	ND	ND	83,3	ND	1329	ND	10,3	ND	ND
ISG8.108	d8	3-21*01	3-3*01	5*02	γ1	DLNDFWSGYQRSGWFDP	17	1	3	7	2,4	κ	3-11*01	2*01	QQRSNWSYT	9	1	0	7	1,9	1	5,7	0,1	5,7	ND	ND	ND	ND	ND	ND	ND	ND	ND	81,4	ND	1241	ND	0	ND	ND
ISG8.109	d8	4-38-2*01	3-10*01	4*02	γ1	HGLLRGGSGGFDY	15	2	1	5	1,7	λ	3-21*03	3*02	QVWDSSSDHWV	11	1	2	4	1,4	1	5,6	0,1	5,6	ND	ND	ND	ND	ND	ND	ND	ND	ND	81,0	ND	1230	ND	0	ND	ND
ISG8.113	d8	3-30*02	4-23*01	3*02	γ1	NYVDNFDI	8	0	2	7	2,4	κ	3-11*01	4*01	HQRSNWPPT	9	2	0	5	1,9	1	5,6	0,1	5,6	ND	ND	ND	ND	ND	ND	ND	ND	ND	74,4	ND	1263	ND	3,4	ND	ND
ISG8.117	d8	3-30*02	3-16*01	3*02	γ1	NYVDAFDI	8	0	2	6	2,0	κ	1-39*01	2*03	QQSYSTPYS	9	0	0	7	2,6	1	5,7	0,1	5,7	ND	ND	ND	ND	ND	ND	ND	ND	ND	76,9	ND	1328	ND	0	ND	ND
ISG8.119	d8	4-61*02	2-2*02	6*03	γ1	ETVVPADIAVRGYYFYYYMDV	21	1	3	3	1,0	λ	1-36*01	1*01	AAWDDSLSGYV	11	0	2	1	0,3	1	5,7	0,1	5,7	ND	ND	ND	ND	ND	ND	ND	ND	ND	81,6	ND	1232	ND	20,4	ND	ND
ISG8.120	d8	3-21*01	3-3*02	4*02	γ1	RSGFSGAEDYHLGY	14	2	2	2	0,7	λ	3-1*01	1*01	QAWDSSTYV	9	0	1	3	1,1	1	5,7	0,1	5,7	ND	ND	ND	ND	ND	ND	ND	ND	ND	70,3	ND	1256	ND	8,1	ND	ND
ISG8.131	d8	4-61*02	3-22*01	4*02	γ1	AHYDSSYYSLGSDY	14	1	2	6	2,0	λ	3-25*02	1*01	QSADSSGTYVV	11	0	1	4	1,4	1	5,7	5,7	0,2	12,2	7,592	11,88	13,07	8,572	12,48	13,13	13,2	13,1	11,1	17,5	1499	1384	38,9	6,8	10,0
ISG8.132	d8	3-49*04	1-1*01	4*02	γ1	VGVHLWPLDY	10	1	1	10	3,3	κ	3-20*01	1*01	QQYGDSPWT	9	0	1	10	3,7	1	5,7	5,7	0,2	0,341	0,272	0,2829	2,013	1,015	3,162	10,85	12,67	13,3	28,0	28,1	1538	1379	18,4	0,0	2,7
ISG8.133	d8	3-21*01	6-19*01	6*03	γ3	DGGLLRIHGLIYYYYMDV	18	1	3	1	0,3	λ	1-51*01	1*01	GTWDSSLRGV	10	1	1	3	1,0	1	5,7	0,1	5,6	ND	ND	ND	ND	ND	ND	ND	ND	ND	82,0	ND	1253	ND	15,0	ND	ND
ISG8.137	d8	3-23*04	2-2*02	5*02	γ3	DGELCSRTNCYTGWFDP	17	1	3	1	0,3	κ	4-69*01	3*02	QTWGTGIRV	9	1	0	6	2,0	1	5,7	0,1	5,7	ND	ND	ND	ND	ND	ND	ND	ND	ND	77,5	ND	1291	ND	ND	ND	ND
ISG8.140	d8	3-21*01	3-3*01	4*02	γ1	EERFLESLSIDY	12	1	4	10	3,4	λ	3-1*01	2*01	QAWDSSTVV	9	0	1	8	2,9	1	5,7	0,1	5,7	ND	ND	ND	ND	ND	ND	ND	ND	ND	83,6	ND	1302	ND	0	ND	ND
ISG8.141	d8	4-39*07	7-27*01	3*02	γ1	SIGGLGMAFDI	11	0	1	6	2,0	κ	1-33*01	4*01	QHFDNLPLT	9	1	1	4	1,6	1	5,7	0,1	5,7	ND	ND	ND	ND	ND	ND	ND	ND	ND	77,3	ND	1295	ND	0	ND	ND
ISG8.143	d8	3-30-3*01	5-12*01	4*02	γ1	DGYSSSFFDY	10	0	2	12	4,1	κ	1-5*03	4*01	QHYNSYSGLT	10	1	0	6	2,3	1	5,7	5,7	0,2	0,3127	0,2684	0,2803	0,357	0,4443	13,04	13,32	13,33	13,3	6,7	6,5	1366	1343	62,7	7,3	11,9
ISG8.151	d8	1-2*02	6-6*01	4*02	γ1	LTTTARQRDY	10	2	1	15	5,1	κ	2-28*01	5*01	MQALQTPIT	9	0	0	3	1,1	2	5,7	5,7	0,2	0,3274	0,308	0,2921	8,17	9,399	12,88	13,31	13,33	13,4	7,4	7,8	1501	1464	93,9	16,7	13,5
ISG8.154	d8	3-21*01	1-26*01	4*02	γ1	DSLFKWDHHFDY	12	3	3	5	1,7	λ	1-44*01	1*01	ATWDDSLNGQV	11	0	2	4	1,4	1	5,7	0,1	5,7	ND	ND	ND	ND	ND	ND	ND	ND	ND	83,6	ND	1332	ND	0	ND	ND
ISG9.101	d9	4-61*02	2-2*02	6*03	γ1	EEPVPAAIRYYFYYYMDV	19	1	3	1	0,3	λ	1-36*01	1*01	AAWDDRLNAYV	11	1	2	3	1,0	1	5,7	0,2	5,7	ND	ND	ND	ND	ND	ND	ND	ND	ND	82,7	ND	1299	ND	0	ND	ND
ISG9.104	d9	4-61*02	5-18*01	6*03	γ1	ETADTAMVSYYFYYYMDV	18	0	3	5	1,7	λ	1-44*01	1*01	AAWDDSLNGYV	11	0	2	2	0,7	1	5,7	0,1	5,7	ND	ND	ND	ND	ND	ND	ND	ND	ND	76,1	ND	1267	ND	1,3	ND	ND
ISG9.105	d9	4-61*02	3-22*01	6*03	γ1	ERNYYDTSGYYSHYYYMDV	19	2	3	4	1,3	λ	1-47*01	1*01	AAWDDSLSGYV	11	0	2	7	2,4	1	5,7	0,1	5,7	ND	ND	ND	ND	ND	ND	ND	ND	ND	75,4	ND	1267	ND	0	ND	ND
ISG9.108	d9	4-61*02	6-19*01	4*02	γ1	AEYSSGWYSGGDDS	14	0	3	9	3,0	λ	3-25*02	3*02	QSADSSGIYWV	11	0	1	10	3,5	2	5,7	5,7	4,2	11,66	12,2	12,53	13,13	11,89	13,12	13,25	13,29	13,1	17,2	42,2	1354	1268	24,1	12,8	4,5
ISG9.110	d9	4-61*10	1-26*01	3*02	γ1	LVGRTFFDY	9	1	1	5	1,7	κ	1-33*01	2*01	QQYDNLPYT	9	0	1	5	1,9	1	5,7	0,1	5,7	ND	ND	ND	ND	ND	ND	ND	ND	ND	81,6	ND	1260	ND	0,3	ND	ND
ISG9.123	d9	3-48*03	5-18*01	3*01	γ1	MPGGYSFGFHAFDV	14	1	1	12	4,1	κ	4-1*01	3*01	QQYYSTPFT	9	0	0	1	0,4	1	5,7	0,1	5,6	ND	ND	ND	ND	ND	ND	ND	ND	ND	83,0	ND	1273	ND	10,6	ND	ND
ISG9.128	d9	3-74*01	3-10*03	4*02	γ1	SGLDY	5	0	1	12	4,1	κ	3-11*01	3*01	QQHSNWPFT	9	1	0	9	3,4	1	5,7	0,1	5,7	ND	ND	ND	ND	ND	ND	ND	ND	ND	76,5	ND	1289	ND	11,6	ND	ND
ISG9.129	d9	3-48*03	5-18*01	3*02	γ1	LPGGYSYGFHAFDI	14	1	1	13	4,4	κ	4-1*01	3*01	QQYYSTPFT	9	0	0	8	2,8	1	5,7	0,2	5,7	ND	ND	ND	ND	ND	ND	ND	ND	ND	76,5	ND	1294	ND	3,4	ND	ND
ISG9.145	d9	3-30*02	3-9*01	4*02	γ1	NLVDNFDL	8	0	2	17	5,8	κ	3-11*01	1*01	QQRSNWPPWT	10	1	0	2	0,7	1	5,7	0,1	5,7	ND	ND	ND	ND	ND	ND	ND	ND	ND	83,5	ND	1307	ND	3,1	ND	ND

Sequence (aa): Red and Blue colors indicate positively and negatively charged amino acids, respectively, in the IgH and IgL complementary determining region (CDR3).
(+) an (-) indicate the numbers of positively and negatively charged amino acids, respectively, in the IgH and IgL complementary determining region (CDR3).
MUT, number of nucleotide somatic mutations.
EC50, half-maximal lethal effective concentration.
CSPR, circumsporozoite precipitation reaction.
ADCP, antibody-dependent cellular phagocytosis.
ND, not determined.

Table S3. SPR single-cycle kinetics (SCK) values of CRR antibodies to CSP18

mAb	k_{on} $(M^{-1}s^{-1})$	k_{off} (s^{-1}) [a]	K_D (pM) [b]	Normalized binding[c]	R_{max} (RU) [d]	χ^2 (RU^2) [e]
ISG3.101	1,51E+07	<0,00001	< 0,66	0,0343	11,5	0,131
ISG3.102	4,15E+06	<0,00001	< 2,40	0,0190	11,4	0,764
ISG3.122	1,43E+07	<0,00001	< 0,70	0,0712	24,1	0,767
ISG3.136	3,29E+07	<0,00001	< 0,30	0,0502	10,9	0,359
ISG4.107	2,07E+07	<0,00001	< 0,48	0,0241	3,9	0,465
ISG4.135	9,01E+07	<0,00001	< 0,11	0,0447	10,3	0,303
ISG4.171	4,32E+07	<0,00001	< 0,23	0,0439	9,7	0,392
ISG5.110	6,77E+06	<0,00001	< 1,50	0,0162	9,9	0,41
ISG5.116	2,42E+07	<0,00001	< 0,41	0,0446	10,3	0,299
ISG5.130	2,32E+07	<0,00001	< 0,43	0,0410	10,3	0,208
ISG5.138	5,91E+07	<0,00001	< 0,17	0,0347	14,1	0,16
ISG5.143	8,77E+07	<0,00001	< 0,11	0,0400	10,6	0,175
ISG5.144	1,20E+07	<0,00001	< 0,83	0,0291	10,6	0,186
ISG5.154	1,13E+07	<0,00001	< 0,88	0,0276	6,8	0,293
ISG5.163	2,74E+07	<0,00001	< 0,36	0,0402	10,4	0,168
ISG5.166	9,55E+06	<0,00001	< 1,00	0,0326	10,1	0,314
ISG5.167	3,47E+07	<0,00001	< 0,29	0,0432	11,8	0,148
ISG5.179	3,89E+06	<0,00001	< 2,60	0,0149	10,9	0,357
ISG6.104	8,48E+06	<0,00001	< 1,20	0,0264	8,5	0,5
ISG6.106	4,61E+06	<0,00001	< 2,20	0,0153	10,3	0,142
ISG6.118	1,48E+07	<0,00001	< 0,68	0,0324	7,1	0,177
ISG6.139	8,87E+06	1,87E-04	21,10	0,0396	23	0,754
ISG6.149	1,22E+07	<0,00001	< 0,82	0,0615	22,3	0,943
ISG6.160	2,61E+07	<0,00001	< 0,38	0,0665	23,3	1,07
ISG6.167	5,86E+06	<0,00001	< 1,70	0,0260	24,1	0,625
ISG6.170	4,49E+06	<0,00001	< 2,20	0,0196	11,6	0,563
ISG8.131	4,43E+06	<0,00001	< 2,30	0,0300	16,9	0,751
ISG8.132	5,78E+06	<0,00001	< 1,70	0,0435	31,9	0,762
ISG8.143	1,36E+07	<0,00001	< 0,74	0,0743	28,2	0,687
ISG8.151	1,20E+07	<0,00001	< 0,83	0,0667	22,6	0,613
ISG9.108	4,62E+06	<0,00001	< 2,20	0,0184	10,3	0,782

[a] The slow dissociation rate precluded an accurate measurement. 0,00001 is the limit of detection of the equipment. [b] KD values expressed with < correspond to the upper limit of picomolar range. [c] The analyte binding level divided by the ligand capture level. This corrects for variations in target density to allow direct comparison of binding efficiency. [d] The maximum response or maximum binding capacity. [e] χ^2 indicates the goodness of the overall modeling; the lower the value, the better is the fit.

Table S4. Affinities of ISG.5.143, ISG5.144, ISG5.163 and ISG6.149 antibodies and their fully germline-reverted variants

	**CSP18**				**$NANP_5$**				**$NANP_3$**			
	k_{on} ($M^{-1}s^{-1}$)	k_{off} (s^{-1})	K_D (M)	χ^2 (RU^2)	k_{on} ($M^{-1}s^{-1}$)	k_{off} (s^{-1})	K_D (M)	χ^2 (RU^2)	k_{on} ($M^{-1}s^{-1}$)	k_{off} (s^{-1})	K_D (M)	χ^2 (RU^2)
ISG5.143	3,7E+07	1,6E-03	4,3E-11	0,1	4,6E+06	4,0E-03	7,4E-10	4,3	n.d	n.d	n.d	n.d
ISG5.143GL	n.d	n.d	n.d	n.d	n.d	n.d	n.d	n.d	n.d	n.d	n.d	n.d
ISG5.144	7,9E+06	6,6E-04	8,3E-11	0,1	9,6E+05	1,3E-03	1,2E-09	0,2	6,2E+05	4,3E-02	7,3E-08	0,9
ISG5.144GL	1,4E+07	3,5E-03	2,5E-10	0,077	3,4E+06	1,4E-02	4,1E-09	0,592	n.d	n.d	n.d	n.d
ISG5.163	5,7E+07	3,5E-03	5,7E-11	0,3	9,2E+06	1,1E-02	1,5E-09	3,1	7,4E+05	9,5E-02	1,3E-07	0,2
ISG5.163GL	1,7E+07	3,7E-03	2,3E-10	0,0923	4,8E+06	5,5E-02	1,1E-08	3	n.d	n.d	n.d	n.d
ISG6.149	1,8E+07	1,1E-03	6,3E-11	0,1	1,9E+06	1,2E-03	6,5E-10	0,7	9,0E+05	2,9E-02	4,5E-08	0,1
ISG6.149GL	2,6E+07	3,5E-03	1,3E-10	0,0563	1,0E+07	1,3E-02	1,3E-09	2,53	n.d	n.d	n.d	n.d

n.d.: not determined due to inaccurate fitting and/or unspecific binding detected by surface plasmon resonance. χ^2 indicates the goodness of the overall modeling; the lower the value, the better is the fit.

Tables S5. CryoEM data collection and refinement statistics for the Fab-rsCSP complexes

	ISG5.143 (EMDB-79206) (PDB 38XB)	ISG5.144 (EMDB-79207) (PDB 38XC)	ISG5.163 (EMDB-79205) (PDB 38XA)	ISG6.149 (EMDB-79208) (PDB 38XD)	ISG6.149GL_HC (EMDB-79210) (PDB 38XF)	ISG6.149GL_LC (EMDB-79209) (PDB 38XE)
**Data collection and processing**						
Magnification	190x	190x	190x	190x	190x	190x
Voltage (kV)	200	200	200	200	200	200
Electron exposure (e–/Å^2)	60	60	60	60	60	60
Defocus range (μm)	-0.8 to -1.8	-0.8 to -1.8	-0.8 to -1.8	-0.8 to -1.8	-0.8 to -1.8	-0.8 to -1.8
Pixel size (Å)	0.725	0.725	0.725	0.725	0.718	0.718
Symmetry imposed	C1	C1	C1	C1	C1	C1
Initial particle images (no.)	725 876	1 007 757	736 123	858 516	1 305 259	1 302 979
Final particle images (no.)	361 978	75 841	51 617	492 414	138 580	409 848
Map resolution (Å)	3.13	3.55	3.39	2.87	3.17	2.90
FSC threshold	0.143	0.143	0.143	0.143	0.143	0.143
**Refinement**						
Map sharpening *B* factor (Å^2)	-100.4	-85.9	-65.3	-75.8	-66.4	-87.2
Model composition						
Non-hydrogen atoms	9200	5425	10790	10773	7112	8960
Protein residues	1191	701	1402	1396	921	1158
B factors (Å^2)						
Protein	49.34	82.92	74.78	83.47	36.93	37.86
R.m.s. deviations						
Bond lengths (Å)	0.004	0.004	0.004	0.003	0.004	0.004
Bond angles (°)	0.630	0.778	0.771	0.644	0.790	0.728
Validation						
MolProbity score	1.66	1.61	1.71	1.67	1.83	1.58
Clashscore	6.85	4.42	5.48	6.25	7.38	5.87
Poor rotamers (%)	0	0	0	0	0	0
Ramachandran plot						
Favored (%)	95.86	94.32	93.81	95.33	93.57	96.2
Allowed (%)	4.14	5.68	6.19	4.67	6.43	3.8
Disallowed (%)	0	0	0	0	0	0

Table S6. Helical parameters of Fab-rsCSP cryo-EM structures

	Rise (Å)		Helical turn (°)	
	Mean	SD	Mean	SD
ISG5.144	6.76825	2.38952595	101.5338	5.19850763
ISG5.163	7.163525	3.76488567	103.57395	8.51480138
ISG5.143	12.23145	0.22274644	85.877	0.54801801
ISG6.149	14.26004	0.14077494	73.94268	0.21377582

Table S7. X-ray data collection and refinement statistics for matured Fabs

	ISG6.149	ISG5.144	ISG5.163
**Data collection**			
Beamline	SSRL 12-1	SSRL 12-1	SSRL 12-1
Wavelength (Å)	0.97946	0.97946	0.97946
Resolution (Å)	50.00-2.25 (2.29-2.25)[a]	50.00-1.99 (2.03-1.99)[a]	50.00-2.21 (2.25-2.21)[a]
Space group	$P2_1$	$P2_12_12_1$	$P2_12_12_1$
Unit cell a, b, c (Å)	90.64, 73.78, 106.16	47.48, 74.51, 135.95	56.95, 71.98, 134.04
α, β, γ (°)	90, 100.9, 90	90, 90, 90	90, 90, 90
Unique reflections	63,026 (3153)[a]	33,416 (1606)[a]	28,292 (1375)[a]
Redundancy	3.5 (3.5)[a]	5.4 (3.1)[a]	6.3 (5.5)[a]
Completeness (%)	96.1 (97.6)[a]	99.2 (97.6)[a]	99.5 (99.9)[a]
Mean I/sigma(σ_I)	18.2 (2.0)[a]	10.8 (1.4)[a]	27.4 (4.8)[a]
R_{sym} (%)[b]	5.5 (50.5)[a]	16.2 (92.9)[a]	11.4 (91.9)[a]
R_{pim} (%)[b]	4.1 (33.9)[a]	9.2 (53.8)[a]	5.6 (30.7)[a]
$CC_{1/2}$ (%)[c]	99.3 (71.4)[a]	98.1 (53.4)[a]	99.1 (84.5)[a]
**Refinement statistics**			
Resolution (Å)	47.54-2.25	44.83-1.99	49.05-2.21
Reflections (work)	62,736	33,273	28,206
Reflections (test)	1,999	1,998	2,000
R_{cryst}[d] / R_{free}[e] (%)	21.2/24.0	19.1/23.8	20.9/24.6
Number of atoms			
Fab	6,610	3,304	3,294
Ligand	138	69	70
Water	101	160	99
Average B-value (Å^2)			
Fab	44	24	32
Water	40	27	22
Wilson B (Å^2)	40	21	12
RMSD from ideal geometry			
Bond angle (°)	0.57	1.15	0.66
Bond length (Å)	0.002	0.011	0.004
Ramachandran statistics[f]			
Favored (%)	98.05	97.24	98.22
Outliers (%)	0.00	0.00	0.00
**PDB Code**	38VS	38VP	38VO

[a] All numbers in parentheses refer to the highest resolution shell.
[b] Rsym = Σhkl Σi | Ihkl,i - | / Σhkl Σi Ihkl,i and R_{pim} = Σhkl (1/(n-1))1/2 Σi | Ihkl,i - | / Σhkl Σi Ihkl,i, where Ihkl,i is the scaled intensity of the ith measurement of reflection h, k, l, is the average intensity for that reflection, and n is the redundancy.
[c] $CC_{1/2}$ = Pearson correlation coefficient between two random half datasets.
[d] R_{cryst} = Σhkl | Fo - Fc | / Σhkl | Fo | x 100, where Fo and Fc are the observed and calculated structure factors, respectively.
[e] R_{free} was calculated as for Rcryst, but on a test set comprising 5% of the data excluded from refinement.
[f] From MolProbity (Williams CJ, et al., (2018). Protein Sci 27:293-315)

Table S8. X-ray data collection and refinement statistics for germline-reverted Fabs

	ISG6.149GL	ISG5.144GL	ISG5.163GL
**Data collection**			
Beamline	SSRL 12-1	SSRL 12-1	SSRL 12-1
Wavelength (Å)	0.97946	0.97946	0.97946
Resolution (Å)	30.00-1.99 (2.03-1.99)[a]	40.00-1.98 (2.03-1.98)[a]	40.00-2.29 (2.35-2.29)[a]
Space group	$P2_1$	$P2_12_12_1$	$P2_12_12_1$
Unit cell a, b, c (Å)	69.99, 123.00, 73.59	71.79, 84.04, 187.44	47.16, 74.83, 141.65
α, β, γ (°)	90, 93.4, 90	90, 90, 90	90, 90, 90
Unique reflections	81,965 (3923)[a]	75,678 (3814)[a]	22,506 (1138)[a]
Redundancy	3.2 (2.8)[a]	6.0 (6.6)[a]	6.1 (6.5)[a]
Completeness (%)	97.2 (93.5)[a]	95.1 (97.2)[a]	96.6 (99.6)[a]
Mean I/sigma(σ_I)	13.3 (1.1)[a]	22.0 (3.9)[a]	36.2 (17.5)[a]
R_{sym} (%)[b]	7.2 (58.2)[a]	6.1 (24.4)[a]	6.1 (11.5)[a]
R_{pim} (%)[b]	6.2 (41.1)[a]	3.3 (13.2)[a]	2.6 (4.7)[a]
$CC_{1/2}$ (%)[c]	98.2 (63.7)[a]	99.9 (97.7)[a]	99.5 (99.0)[a]
**Refinement statistics**			
Resolution (Å)	29.91-1.99	39.24-1.98	30.48-2.29
Reflections (work)	81,684	75,192	22,427
Reflections (test)	2,004	1,998	2,000
R_{cryst}[d] / R_{free}[e] (%)	20.2/22.6	20.5/24.8	20.8/25.6
Number of atoms			
Fab	6,566	6,636	3,290
Ligand	138	138	61
Water	322	333	154
Average B-value (Å^2)			
Fab	34	31	18
Water	35	32	20
Wilson B (Å^2)	32	27	17
RMSD from ideal geometry			
Bond angle (°)	0.75	0.83	0.64
Bond length (Å)	0.005	0.006	0.003
Ramachandran statistics[f]			
Favored (%)	97.56	97.94	98.15
Outliers (%)	0.00	0.00	0.00
**PDB Code**	38VR	38VQ	38VN

[a] All numbers in parentheses refer to the highest resolution shell.
[b] Rsym = Σhkl Σi | Ihkl,i - | / Σhkl Σi Ihkl,i and R_{pim} = Σhkl (1/(n-1))1/2 Σi | Ihkl,i - | / Σhkl Σi Ihkl,i, where Ihkl,i is the scaled intensity of the ith measurement of reflection h, k, l, is the average intensity for that reflection, and n is the redundancy.
[c] $CC_{1/2}$ = Pearson correlation coefficient between two random half datasets.
[d] R_{cryst} = Σhkl | Fo - Fc | / Σhkl | Fo | x 100, where Fo and Fc are the observed and calculated structure factors, respectively.
[e] R_{free} was calculated as for Rcryst, but on a test set comprising 5% of the data excluded from refinement.
[f] From MolProbity (Williams CJ, et al., (2018). Protein Sci 27:293-315).
